# Identification of specific microbiota members that induce beige fat biogenesis in response to dietary cues

**DOI:** 10.21203/rs.3.rs-5454144/v1

**Published:** 2024-12-03

**Authors:** Kenya Honda, Takeshi Tanoue, Manabu Nagayama, Ayumi Roochana, Samuel Zimmerman, Orr Ashenberg, Tanvi Jain, Satoshi Sasajima, Kozue Takeshita, Nicola Hetherington, Nobuyuki Okahashi, Masahiro Ueda, Morichika Konishi, Yoshiaki Nakayama, Aki Minoda, Ashwin Skelly, Yasuhiko Minokoshi, Nicholas Pucci, Daniel Mende, Makoto Arita, Hironori Yamamoto, Shunji Watanabe, Kouichi Miura, Wataru Suda, Koji Atarashi, Mami Matsushita, Shingo Kajimura, Damian Plichta, Masayuki Saito, Ramnik Xavier

**Affiliations:** Keio University School of Medicine; Keio University School of Medicine; Keio University School of Medicine; Keio University School of Medicine; Infectious Disease and Microbiome Program, Broad Institute of MIT and Harvard; Broad Institute of MIT and Harvard; Klarman Cell Observatory, Broad Institute of MIT and Harvard; Keio University School of Medicine; Keio University School of Medicine; RIKEN Center for Integrative Medical Sciences; RIKEN; RIKEN Center for Integrative Medical Sciences; Kobe Pharmaceutical University; Laboratory of Microbial Chemistry, Kobe Pharmaceutical University; RIKEN; Keio University School of Medicine; National Institute for Physiological Sciences; Department of Medical Microbiology, Amsterdam UMC, University of Amsterdam; Department of Medical Microbiology, Amsterdam UMC, University of Amsterdam; RIKEN Center for Integrative Medical Sciences; Division of Gastroenterology, Department of Medicine, Jichi Medical University; Division of Gastroenterology, Department of Medicine, Jichi Medical University; Division of Gastroenterology, Department of Medicine, Jichi Medical University; RIKEN Center for Integrative Medical Sciences; Keio University School of Medicine; Tenshi College; Beth Israel Deaconess Medical Center, Harvard Medical School, and Howard Hughes Medical Institute; Broad Institute; Hokkaido University; Massachusetts General Hospital

## Abstract

Activation of brown and beige fat biogenesis promotes metabolic health in rodents and humans, but typically requires cold exposure or pharmacological activation of β-adrenergic receptors, which may pose cardiovascular risks. Dietary intervention represents a clinically viable alternative strategy to induce beige cells and thus enhance metabolic health, though the underlying mechanisms remain poorly understood. In this study, we identified specific microbiota members in both mice and humans that promote browning of white adipose tissue (WAT) and ameliorate metabolic disorders in the context of a low-protein diet (LPD). Diets with low protein, regardless of fat and carbohydrate content, induced robust WAT browning to a degree comparable to that achieved by cold exposure or β-adrenergic receptor agonist administration. LPD-mediated browning was markedly diminished in germ-free (GF) mice, highlighting the essential role of the gut microbiota. Microbiota-induced browning occurred independently of the immune system and primarily through mechanisms involving increased circulating deconjugated bile acids, activation of the farnesoid X receptor (FXR) in WAT progenitor cells, and enhanced hepatic production of FGF21. The browning defect in GF mice was rescued by transplanting microbiota from conventional mice or from brown/beige fat-positive healthy human volunteers, as determined by fluorodeoxyglucose positron-emission tomography (FDG-PET). Defined bacterial consortia, comprising strains isolated from mice or FDG-PET-positive human volunteers, were sufficient to elevate plasma bile acids and hepatic FGF21 levels by modulating nitrogen metabolism, ultimately restoring browning in response to a LPD. Our findings highlight the significant impact of diet-microbiota interactions on WAT browning and suggest their therapeutic potential for managing metabolic diseases.

Excessive lipid storage in WAT and the resulting inflammation are intimately linked to metabolic dysfunction. In contrast, thermogenic adipocytes, namely brown and beige cells, are protective against cardiometabolic diseases. Recent studies identified external cues, including chronic cold exposure, catecholamine stimulation, caloric restriction, and exercise, that promote beige fat biogenesis (also referred to as WAT “browning” or “beiging”) by facilitating white-to-beige conversion as well as beige progenitor cell proliferation^6–14^. The emerged beige adipocytes are enriched with mitochondria and express a diverse array of genes involved in thermogenesis and fatty acid β-oxidation, such as uncoupling protein 1 (*UCP1*), elongation of very long chain fatty acid 3 (*ELOVL3*) and cell death inducing DFFA like effector A (C/ZEA)^9,10,12,15,16^. Accordingly, the induction and activation of beige adipocytes have beneficial effects on reducing adiposity and mitigating metabolic diseases^9,12^. Notably, beige-like inducible adipocytes are found in human adipose tissue, and can be identified as FDG-PET scan-positive spots in the supraclavicular and abdominal regions^17–21^.

While chronic cold exposure and administration of catecholamine receptor agonists have been shown to effectively induce beige adipocyte biogenesis, these approaches are not suitable for therapeutic use due to undesirable cardiovascular risks. Diet-based interventions represent a promising alternative, as previous studies have shown that caloric restriction, methionine or leucine deprivation, and ketogenic diets activated BAT and induced WAT browning^22–27^. Additionally, the gut microbiota has been implicated in the regulation of browning, potentially via production of bioactive metabolites^28–32^ and immunomodulation^33,34^. However, the mechanisms by which diet-microbiota interplay promotes WAT browning, as well as the specific bacterial strains involved, remain incompletely understood.

## Low-protein diets promote beige fat biogenesis

**Figure 1:**
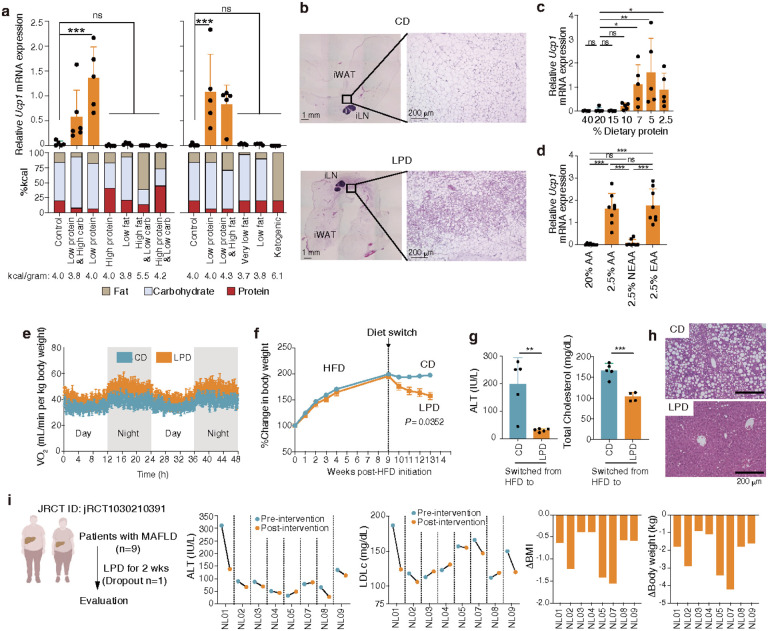
Low protein diets induce beige cells and ameliorate obesity and fatty liver disease. **a,** SPF B6 male mice were fed diets with varying proportions of protein, carbohydrates, and fats for 6 weeks. *Ucp1* mRNA expression in inguinal white adipose tissue (iWAT), normalized to *Ppib,* is shown along with stacked bar graphs representing each diet’s macromolecular composition and total energy content (kcal/g). The left and right panels show results from two independent experiments. **b,** Hematoxylin and eosin (H&E) staining of iWAT from mice fed either a control diet (CD, 20% protein content) or a low-protein diet (LPD, 7% protein content) for 6 weeks. **c, d,** Relative iWAT *Ucp1* expression in mice fed isocaloric diets containing various concentrations of protein **(c)** or 2.5% essential or nonessential amino acids **(d)** for 6 weeks. **e,** Oxygen consumption rate (VO_2_) of SPF B6 mice fed a CD or LPD (n=5 mice per group) was measured every 3 minutes over 48 hours. Mean ± s.e.m. is shown at each time point. **f-h,** SPF B6 male mice were fed a high-fat diet (HFD) for 9 weeks and then switched to a CD or LPD (n=5 mice per group). Body weight change **(f),** plasma levels of alanine transaminase (ALT) and cholesterol **(g),** and representative liver H&E staining **(h)** are shown. i, Patients with fatty liver disease were placed on commercially available LPDs for 2 weeks. Changes in plasma ALT, LDL(low-density lipoprotein cholesterol), body mass index (BMI), and body weight are shown. In **a, c, d, g,** each circle represents an individual animal, and the height of each bar represents the mean ± s.d. ***P < 0.001; **P < 0.01; *P < 0.05; ns, not significant; analyzed by one-way ANOVA with Benjamini–Hochberg correction for multiple comparisons **(a, d),** Mann-Whitney test (two-tailed) for each comparison **(c),** unpaired t-test **(g),** or two-way ANOVA with Bonferroni’s correction **(f).**

We set out to rigorously investigate the impact of dietary modifications on WAT browning. To do so, we formulated diets with varying proportions of protein, carbohydrates, and fats. While changes in fat content affected the total calorie count, all other diets were designed to be isocaloric. These diets were administered to specific pathogen-free (SPF) C57BL/6 (B6) mice housed at room temperature (25°C). Regardless of lipid and carbohydrate content, feeding mice diets with low protein content significantly increased *Ucp1* mRNA expression in the inguinal WAT (iWAT) (Fig. 1a). H&E staining of iWAT from mice fed a low protein diet (LPD) revealed features characteristic of browning, including eosin-dense staining of cells with multilocular lipid droplets, most notably in the central area adjacent to the inguinal lymph node (Fig. 1b). The effect of a LPD was robust, as including either high-fat or high-carbohydrate contents did not affect LPD-induced browning effect. Although previous studies have suggested that very-low-carbohydrate ketogenic diets activate BAT^27^, a ketogenic diet did not affect *Ucp1* expression in iWAT in our hands (Fig. 1a). LPD-driven beige induction in iWAT was not specific to B6 mice but was commonly observed in mice of different genetic backgrounds including BALB/c and ICR (**Extended Data Fig. 1a**). However, aged or female B6 mice displayed a relative resistance to LPD-induced browning compared to young or male mice, respectively (**Extended Data Fig. 1b**). Additionally, unlike iWAT, perigonadal WAT (gWAT) from B6 mice did not exhibit increased *Ucp1* expression (**Extended Data Fig. 1c**). These results suggest that iWAT in older or female mice and gWAT in B6 mice may contain fewer or functionally distinct adipocyte precursor cells^11,35^. LPD feeding also led to an increase in *Ucp1* expression in interscapular BAT, although the fold change from baseline was lower compared to that observed in iWAT (**Extended Data Fig. 1d**).

We next examined the duration of LPD required for beige cell induction. *Ucp1* expression was detected as early as 2 weeks post-LPD initiation and continued to increase thereafter, plateauing by week 6–8 (**Extended Data Fig. 1e**). To determine the threshold at which low dietary protein induces browning, B6 mice were fed diets containing varying levels of proteins. Diets with ≤ 7% protein content (approximately 60% less than the control AIN93G diet) strongly induced *Ucp1* expression in iWAT (Fig. 1c), which was confirmed by Western blot and immunostaining analyses (**Extended Data Fig. 1f**). Feeding B6 mice a diet containing 7% protein for 6 weeks did not grossly alter appearance or behavior (**Extended Data Video 1**), indicating that this LPD is well-tolerated and that the observed *Ucp1* upregulation is not attributable to a pathogenic response. Therefore, we selected a 7% protein, 6-week regimen as the standard diet for subsequent LPD experiments, unless otherwise specified. Following the switch back to a regular diet, there was a decline in *Ucp1* mRNA transcripts and cells with beige morphology (**Extended Data Fig. 1g**), indicating that the LPD induces beige cells in a manner that is both inducible and reversible, consistent with previous reports showing the dynamic nature of the browning program^36,37^.

As the type and digestibility of dietary protein may influence phenotype, we next investigated the effects of diets containing defined amino acids instead of natural proteins. Decreasing dietary amino acid content led to a marked elevation in *Ucp1* expression in iWAT (**Extended Data Fig. 2a**). Maximal *Ucp1* expression, comparable to that induced by a 7% LPD, was achieved when the amino acid content was reduced to ≤5%. To investigate which specific amino acids influence iWAT browning, we formulated diets with decreased levels of essential amino acids (EAAs) or non-essential amino acids (NEAAs). Reducing EAAs induced *Ucp1* expression in iWAT to the same extent as did limiting total amino acid content, whereas restricting NEAAs had no discernable effect (Fig. 1d). To determine which of the nine EAAs were responsible, we examined the effect of reducing each individual EAA in the diet. *Ucp1* expression was slightly elevated when dietary isoleucine, leucine, phenylalanine, tryptophan, lysine, or threonine was reduced, though there was substantial variability among mice in each group and none recapitulated the robust *Ucp1* induction observed when total EAAs were limited (**Extended Data Fig. 2b**). Despite previous studies indicating that restricting dietary methionine induces WAT browning^22^, mice on a methionine-reduced diet exhibited only a slight increase in *Ucp1* expression in our hands. Together, these results suggest that reducing individual EAAs may activate distinct pathways, producing a cumulative browning effect.

## Low-protein diets ameliorate obesity and fatty liver

RNAseq and qPCR revealed that a LPD induced upregulation of a broad spectrum of beige signature genes in iWAT (**Extended Data Fig. 2c, d**). Specifically, genes involved in mitochondrial function, thermogenesis, and lipid metabolism, such as *Ucp1, Elovl3, Cidea,* cytochrome c oxidase subunit VIIa polypeptide 1 (*Cox7a1*), and cytochrome c oxidase subunit VIIIb (*Cox8b*)^6^, were significantly upregulated in LPD-fed mice compared to those on a control diet. Glycogenic beige markers were elevated as well, including *Eno1, Ldhb,* and *Pdha*^38^, as was *Phospho1,* a factor implicated in creatine metabolism for UCP1-independent energy expenditure^39^ (**Extended Data Fig. 2d**). These findings suggest that a LPD likely activates multiple signaling pathways, thereby inducing functionally heterogeneous cell subsets including lipolytic, glycogenic, and creatine-metabolizing beige cells.

We next explored the effect of a LPD on energy metabolism in B6 mice using metabolic cages. Compared to control diets, a LPD resulted in higher oxygen consumption, a lower respiratory quotient (indicating enhanced fatty acid β-oxidation), and increased locomotor activity during both day and night (Fig. 1e**, Extended Data Fig. 3a**). These results suggest that the LPD not only induces a metabolic shift towards fat oxidation, but also stimulates physical activity, potentially contributing to changes in energy expenditure. Indeed, despite identical or increased food intake compared to those on control diets (**Extended Data Fig. 3a**), mice on a LPD exhibited significantly reduced body weight and increased glucose tolerance (**Extended Data Fig. 3b**).

We thus investigated the impact of LPD feeding on metabolic disease models, starting with a high-fat diet (HFD)-induced obesity and fatty liver model. B6 mice were fed a HFD for 9 weeks and then switched to either a low-protein or control diet. The LPD led to a significant reduction in body weight, plasma alanine aminotransferase (ALT), and cholesterol levels (Fig. 1f, 1g). Histological examination confirmed that the LPD markedly decreased lipid accumulation in the liver (Fig. 1h). The effects of a LPD were further evaluated in a separate mouse model of metabolic dysfunction induced by a diet high in fat, fructose, and cholesterol [a metabolic dysfunction-associated steatohepatitis (MASH)-inducible diet^40^]. The MASH diet resulted in obesity, impaired glucose tolerance, dyslipidemia, and excess fat accumulation in the liver. Notably, shifting to a LPD markedly ameliorated these symptoms (**Extended Data Fig. 4a-d**).

To investigate the effects of LPDs on human metabolic disease, we conducted a clinical study involving 9 adult patients diagnosed with fatty liver disease, confirmed by ultrasonography and transient elastography^41^. The cohort consisted of 4 women and 5 men, aged 36 to 64 years old. Given the single-arm design and small sample size of the trial, participants were recruited without gender- or age-based restrictions. Participants were placed on commercially-available LPDs, consuming 0.6 to 0.8 grams of protein per kilogram of body weight per day for 2 weeks. Eight participants reported successful completion of the study. Post-intervention analysis revealed substantial interindividual variability; however, patients generally showed reductions in BMI, body weight, and plasma ALT and low-density lipoprotein (LDL) levels compared to baseline measurements, consistent with our mouse studies (Fig. 1i). In summary, while further studies are needed to determine whether LPDs induce beige cell accumulation in humans and the extent to which WAT browning mediates the beneficial effects observed in mice, LPDs hold promise as a therapeutic strategy for metabolic disorders like fatty liver disease.

## Microbiota-dependent browning induced by LPDs

**Figure 2:**
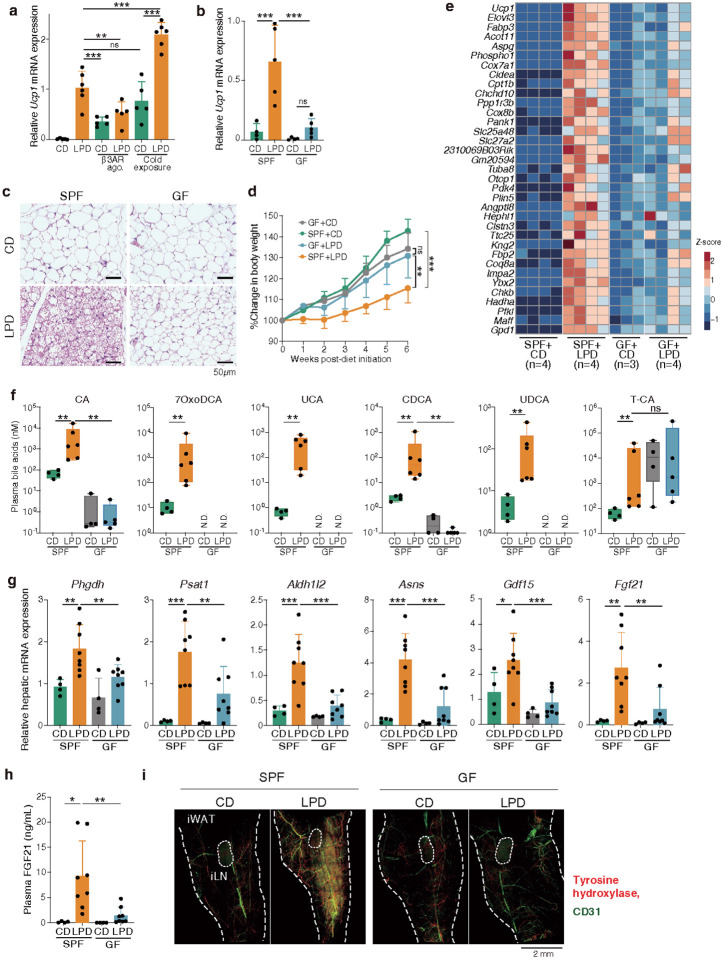
LPD-induced browning is dependent on the microbiota **a,** SPF B6 male mice were fed a CD or LPD for 6 weeks. During the last week, they either received daily intraperitoneal injections of a β3 adrenergic receptor agonist (β3AR ago, CL316,243, 20 mg/mouse/day) or were housed at 6°C. **b-h,** GF and SPF B6 male mice were fed a CD or LPD for 6 weeks. Relative gene expression in iWAT and liver was measured by qPCR and normalized to *Ppib* **(a, b, g).** Representative H&E staining of iWAT **(c)** and percent change in body weight **(d)** is shown. Heat map displays genes with higher expression levels in iWAT from SPF+LPD mice compared to SPF+CD, GF+CD, and GF+LPD mice, as determined by RNA-seq. These genes exhibit RPKM values ≥ 200, a fold change ≥ 4, and an adjusted p-value < 0.05 relative to the SPF+CD condition **(e).** Plasma bile acids were quantified by LC-MS/MS **(f),** relative expression of hepatic genes was quantified by qPCR and normalized to *Ppib* **(g),** and plasma FGF21 was quantified by ELISA **(h).** Representative merged images of whole-mount iWAT staining with anti-tyrosine hydroxylase and anti-CD31 antibodies obtained using a light sheet microscope after iDISCO processing **(i).** Dashed lines indicate the boundaries of the inguinal lymph nodes (center; iLN) and iWAT (periphery). In **a, b, g,** each circle represents an individual animal, and bars indicate the mean ± s.d. In **f,** horizontal lines in the box plots represent the median; box boundaries indicate the interquartile range; and whiskers indicate the data range. ***P < 0.001; **P < 0.01; *P < 0.05; ns, not significant; analyzed by one-way ANOVA with the Benjamini–Hochberg correction for multiple comparisons **(a, b, g, h),** two-way ANOVA **(d),** or two-tailed Mann-Whitney test for each comparison **(f).** N.D., not detected.

We next aimed to explore the mechanistic underpinnings of LPD-mediated beige fat biogenesis. We compared the effectiveness of a LPD to standard browning induction regimens, including chronic cold exposure and catecholamine stimulation. Feeding mice a LPD resulted in robust *Ucp1* upregulation, comparable to that induced by cold exposure (housing at 6°C for 7 days) or intraperitoneal administration of a β3 adrenergic receptor agonist (daily injection of CL316,243 for 7 days) (Fig. 2a). Notably, combining LPD feeding with cold exposure resulted in an additive increase in *Ucp1* expression (Fig. 2a), suggesting that distinct, though possibly overlapping, mechanisms of browning may be at play.

Various immune cell subsets, particularly type 2 innate lymphoid cells (ILC2s), γδ T cells, macrophages, and eosinophils, have been implicated in cold stress-induced WAT browning^42–48^. To assess their role in LPD-mediated browning, we examined a series of mutant mice deficient in various components of the immune system. *Rag2*^−*/−*^*ll2rγ*^−*/−*^ mice, which lack both innate and adaptive lymphocytes, exhibited unimpaired beige fat biogenesis when fed a LPD (**Extended Data Fig. 5**). Similarly, LPD-mediated browning was not affected in *Tcrβ*^−*/−*^*Tcrδ*^−*/−*^ mice (deficient in T cells), *Tbx21*^−*/−*^ mice (deficient in type 1 immunity), *Rorc*^−*/−*^ mice (deficient in type 17 immunity), *II4*^−*/−*^*, II33*^−*/−*^, or *l/5Cre;RosaDTA* mice (deficient in type 2 immunity), *LysM*-Cre;*Rosa*-DTA mice (deficient in myeloid cells), and *Lta*^−*/−*^ mice (deficient in lymphoid tissues) (**Extended Data Fig. 5**). Therefore, while the immune system could still play a role in LPD-mediated browning, we shifted our focus to alternative mechanisms.

Given the growing appreciation for the gut microbiome as a key regulator of host metabolism and dietary responses, we next compared LPD-induced browning in SPF and GF mice. Compared to SPF mice, GF mice exhibited significantly reduced *Ucp1* expression (Fig. 2b) and histological indicators of iWAT browning following LPD treatment (Fig. 2c). Additionally, while a LPD suppressed weight gain in SPF mice, there was no effect in GF mice (Fig. 2d). RNA sequencing of iWAT from GF mice after 6 weeks on the LPD revealed reduced expression of a wide range of beige signature genes, including *Ucp1, Cidea*, *Elovl3, Cox7a1, Cox8b, Clstn3,* and *Acot11* (refs.^11,49,50^) (Fig. 2e). Gene ontology (GO) enrichment analysis highlighted reduced expression of genes involved in lipid metabolism and response to temperature stimuli in LPD-fed GF versus SPF mice (**Extended Data Fig. 6a**). Similar trends were observed in mice fed a low EAA diet (**Extended Data Fig. 6b**). Treating adult SPF mice with an antibiotic cocktail also led to a significant reduction in *Ucp1, Elovl3,* and *Cox7a1* expression in iWAT (**Extended Data Fig. 6c**). These findings indicate that the microbiota plays an essential role in LPD-mediated beige induction.

## Low protein diets alter circulating bile acids

**Figure 3:**
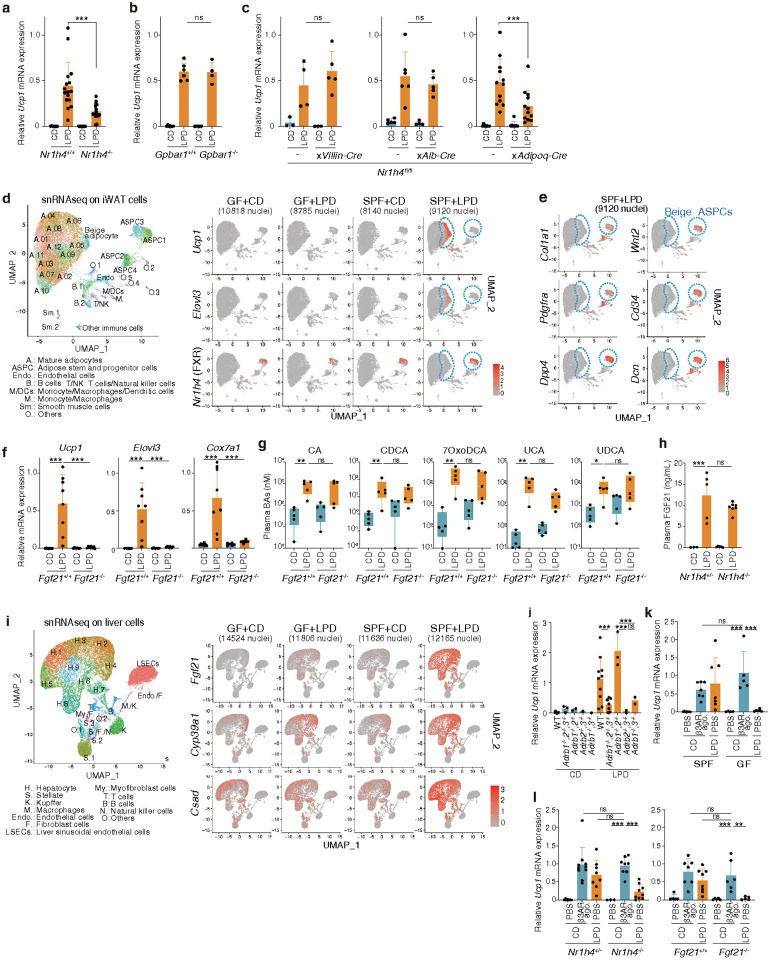
FXR and FGF21 signalling mediate LPD-induced browning. **a–c, f, j,** SPF B6 mice of the indicated genotypes were fed a CD or LPD for 6 weeks. Gene expression in iWAT, normalized to *Ppib,* was measured by qPCR. **d, e, i,** snRNA-seq was performed on iWAT **(d, e)** and liver samples **(i)** from GF and SPF mice fed a CD or LPD. Uniform manifold approximation and projection (UMAP) plots and expression of the indicated genes are shown. **g, h,** Mice of the indicated genotypes were fed a CD or LPD, and plasma bile acids **(g)** and FGF21 **(h)** were quantified by LC-MS/MS and ELISA, respectively. **k, l**, SPF and GF B6 mice, or SPF mice of the indicated genotypes, were fed a CD or LPD for 6 weeks. During the last week, they received daily intraperitoneal injections of a β3 adrenergic receptor agonist (β3AR ago., CL316,243, 20 μg/mouse/day) or PBS. Relative *Ucp1* expression in iWAT, normalized to *Ppib,* was determined by qPCR. Each circle represents an individual animal. In **a–c, f, h, j–l,** the height of each bar indicates the mean ± s.d. Horizontal lines in box plots **(g)** indicate the median; box boundaries indicate the interquartile range; and whiskers indicate the data range. ***P < 0.001; **P < 0.01; *P < 0.05; ns, not significant; analyzed by one-way ANOVA with Benjamini–Hochberg correction for multiple comparisons **(a–c, f, h, j–l)** or two-tailed Mann-Whitney test for each comparison **(g).**

Next, we searched for microbiota-derived molecules that might contribute to iWAT browning. Non-targeted liquid chromatography-mass spectrometry (LC-MS) analysis of ileal and plasma samples revealed that a LPD increased plasma levels of linoleic acid derivatives, such as conjugated linoleic acids (CLA1 and CLA3) and 10-hydroxy-octadecanoic acid (HYB) (**Extended Data Fig. 7a**), consistent with previous reports that linoleic acid metabolism promotes the proliferation of beige fat progenitor cells^14^. Additionally, unconjugated bile acids, including cholic acid (CA) and muricholic acid (MCA), were elevated in the plasma (**Extended Data Fig. 7a**). Targeted LC-MS analysis confirmed increased levels of unconjugated primary bile acids, including CA, aMCA, bMCA, and chenodeoxycholic acid (CDCA). 7α- and 7β-dehydroxylation products^51^, including 7-oxo-deoxycholic acid (7oxoDCA), ursocholic acid (UCA), and ursodeoxycholic acid (UDCA), were also elevated in LPD-fed SPF mice compared to GF and control diet-fed SPF mice (Fig. 2f**, Extended Data Fig. 7b**). While these heightened bile acid concentrations were reproducibly observed in the plasma, no such trends were noted in the intestinal contents (**Extended Data Fig. 7c**). The aforementioned bile acids exerted agonistic activity in an *in vitro* reporter assay for farnesoid X receptor (FXR), one of the primary nuclear receptors activated by bile acids (**Extended Data Fig. 8a**), and as such we next investigated whether FXR signaling was involved in browning. FXR (encoded by *Nrlh4*)*-*deficient mice that were fed a LPD exhibited a consistent, significant reduction in *Ucp1* induction and morphologically beige cells in iWAT (Fig. 3a**, Extended Data Fig. 8b**). We also examined the role of another prominent bile acid receptor, TGR5 (encoded by *Gpbar1*). Despite several reports implicating TGR5-mediated bile acid signaling in WAT browning^30,52^, we found that *Gpbar1*^*−*/−^ mice exhibited comparable browning responses to those observed in wild-type controls (Fig. 3b**, Extended Data Fig. 8c**). These results suggest that bile acids produced by the gut microbiota in response to a LPD promote beige cell induction in an FXR-dependent, TGR5-independent manner, though we cannot exclude involvement of additional bile acid receptors.

Given that FXR is expressed in various organs including the intestine, liver, and WAT^53^, we proceeded to investigate the specific contribution of FXR signaling in each of these tissues. We crossed *NrV1h4*^fl/fl^ mice with Villin-Cre, Albumin-Cre, or Adipoq-Cre mice, allowing targeted FXR deletion in intestinal epithelial cells, hepatocytes, or adipocytes, respectively. When fed a LPD, both *NrV1h4*^fl/fl^;Villin-Cre and *NrV1h4*^fl/fl^;Albumin-Cre mice showed comparable Ucp1 induction to FXR-sufficient controls. In contrast, *NrV1h4*^fl/fl^;Adipoq-Cre mice demonstrated significantly reduced *Ucp1* expression (Fig. 3c) and fewer morphologically beige cells in the iWAT (**Extended Data Fig. 8d**). Together, these findings suggest that the gut microbiota elevates circulating bile acid levels in response to low protein intake, which in turn activates FXR primarily in adipose tissue, thereby promoting beige cell induction.

To identify cell types that express FXR in iWAT, we performed single-nuclear RNA sequencing (snRNA-seq) analysis on iWAT cell nuclei from GF and SPF mice fed a control or low-protein diet. As expected, the putative beige cell cluster expressing *Ucp1* and *Elovl3* was almost exclusively detected in LPD-fed SPF mice (Fig. 3d). Among the reported bile acid receptors^53,54^, pregnane X receptor (PXR, *Nr1i2*), vitamin D receptor (VDR), constitutive androstane receptor (CAR, *Nr1i3*), and TGR5 were not expressed by iWAT cells, whereas liver X receptor alpha (LXR, *Nr1h3*) was broadly expressed by several adipose clusters (**Extended Data Fig. 9a**). Notably, FXR expression exhibited a more restricted pattern, localizing to a cell cluster distinct from mature adipocytes (Fig. 3d). The FXR-expressing cells are likely adipose stem/progenitor cells (ASPCs), as they co-expressed ASPC marker genes including *Col1a1, Pdgfra, Dpp4, Wnt2, Cd34,* and *Dcn*^55–60^ (Fig. 3e**, Extended Data Fig. 9a**). These findings suggest that microbiota-derived bile acids activate FXR in ASPCs, promoting their differentiation into beige cells. Using our snRNA-seq dataset, we performed trajectory inference analysis with Slingshot on adipocytes in LPD-fed SPF mice. Our analysis focused on mature adipocytes and beige cells, as intermediate cell populations between ASPCs and mature adipocytes were not captured. Slingshot predicted transitions linking mature adipocyte clusters, starting from cluster Adipocyte04, to the beige adipocyte cluster (**Extended Data Fig. 9b, c**). The genes encoding carnitine palmitoyltransferase 1 (*Cpt1*), oxoglutarate dehydrogenase (*Ogdh*), pyruvate dehydrogenase kinase 4 (*Pdk4*), acetyl-coenzyme A carboxylase alpha (*Acaca*), Rho GTPase activating protein 26 (*Arhgap26*), and ELOVL fatty acid elongase 6 (*Elovl6*) were sequentially upregulated along pseudotime before *Ucp1* expression in fully-differentiated beige cells (**Extended Data Fig. 9d**). These genes are involved in mitochondrial lipid metabolism and BAT activity^61–65^, suggesting that these pathways are successively acquired during beige cell differentiation in response to a LPD.

## Gut microbiota-dependent FGF21 induction in the liver

Given the robust nature of LPD-induced browning, we hypothesized that multiple, complex pathways may be involved in addition to the bile acid-FXR axis. Considering the central role of the liver in regulating metabolic response to different diets, we performed bulk RNA sequencing and qPCR on liver samples. Several genes were found to be consistently upregulated in SPF mice fed a LPD for 1 or 6 weeks, as compared to those on a control diet or GF mice (**Extended Data Fig. 10**). Upregulated genes included 3-phosphoglycerate dehydrogenase (*Phgdh*), phosphoserine aminotransferase 1 (*Psat1*), aldehyde dehydrogenase 1 family member L2 (*Aldh1l2*), methylenetetrahydrofolate dehydrogenase 2 (*Mthfd2*), and asparagine synthetase (*Asns*) (Fig. 2g**, Extended Data Fig. 10**). PHGDH and PSAT1 have been implicated in the *de novo* synthesis of serine from glycolytic intermediate 3-phosphoglycerate, and MTHFD2 and ALDH1L2 play crucial roles in mitochondrial folate one-carbon metabolism, a major pathway for NADPH production that fuels nucleotide, amino acid, and lipid biosynthesis. ASNS utilizes ammonia and catalyzes the conversion of aspartate to asparagine, which serves as a vital nitrogen reservoir and facilitates nitrogen assimilation, recycling, and remobilization, especially during periods of nitrogen scarcity^66^. These findings suggest that the intestinal microbiota is instrumental in linking dietary modifications to substantial alterations in liver metabolism, characterized as the integrated stress response (ISR) and nitrogen recycling^67^. The aforementioned genes are known to be regulated by activating transcription factor 4 (ATF4)^68,69^. Consistently, other ATF4-inducible genes, such as growth differentiation factor 15 (*Gdf15*), nuclear protein transcription regulator 1 (*Nupr1*), and ChaC cation transport regulator 1 (*Chac1*) were also upregulated in the liver in a microbiota-dependent manner following LPD feeding (Fig. 2g**, Extended Data Fig. 10**), suggesting that hepatic ATF4-inducible genes may play a role in mediating iWAT browning. Importantly, the expression of these genes was generally not altered by administration of β3 adrenergic receptor agonist CL316,243 (**Extended Data Fig. 10**), highlighting the unique nature of LPD-induced tissue signaling in promoting iWAT browning.

Notably, fibroblast growth factor 21 (*Fgf21*), another ATF4-inducible gene^70^, was markedly upregulated in the liver of SPF, but not GF, mice as early as one week post-LPD initiation (Fig. 2g**, Extended Data Fig. 10**). Consistently, FGF21 concentration in the serum was elevated in LPD-fed SPF mice, but not in GF or control diet-fed mice (Fig. 2h). As FGF21 has been reported to stimulate thermogenesis and improve metabolic health^71–75^, we investigated its role in LPD-mediated browning by examining *Fgf21*-deficient mice. *Fgf21*^−*/−*^ mice exhibited a phenotype similar to that of GF mice, with severely suppressed expression of *Ucp1* and other beige markers in iWAT when fed a LPD (Fig. 3f). These findings suggest that FGF21 is essential for LPD-mediated browning and that the gut microbiota plays a role in modulating FGF21 expression in response to dietary changes. Notably, LPD feeding promoted similar plasma bile acid profiles in *Fgf21*^−*/−*^ and WT mice (Fig. 3g), and conversely, similar plasma FGF21 levels in *Nr1h4*^−*/−*^ and WT mice (Fig. 3h). These findings suggest that FXR and FGF21 function through parallel pathways that together promote beige cell induction in iWAT.

To identify which cell types express *Fgf21* in the liver, we conducted scRNA-seq. Robust *Fgf21* expression was detected in hepatocyte clusters from LPD-fed SPF mice, but not in endothelial, Kupffer, or stellate cell clusters, or in any cell subset from GF or control diet-fed mice (Fig. 3i). A similar expression pattern was observed for other ATF4-inducible genes (**Extended Data Fig. 11**) as well as *Cyp39a1* and cysteine sulfinic acid decarboxylase (*Csad*) (Fig. 3i). CYP39A1 is involved in a non-canonical bile acid biosynthetic pathway originating from cholesterol^76^, and CSAD plays a critical role in taurine synthesis, contributing to the production of tauro-CA and tauro-CDCA/MCA^77^. These results suggest that CYP39A1 and CSAD may work in concert within hepatocytes to expand the bile acid pool. Overall, the coordinated hepatic response to diet-conditioned microbiota appears to play a crucial role in the induction of beige adipocytes.

## Activation of sympathetic signalling by a LPD

Cold exposure activates the sympathetic nervous system, leading to norepinephrine release and subsequent browning. Given the established role of catecholamine signaling in browning^8–12,15,78^, we assessed LPD-mediated beige cell induction in mice deficient in β1 (*Adrb1*), β2 (*Adrb2*), and β3 (*Adrb3*) adrenergic receptors. All mice lacking *Adrb3,* including *Adrb1/2/3* triple knockout, *Adrb1/3* double knockout, and *Adrb2/3* double knockout mice, exhibited severe a defect in beige cell induction (Fig. 3j). In contrast, the absence of *Adrb1* and *Adrb2* did not impair beige cell induction, suggesting that LPD-induced browning relies specifically on β3 adrenergic receptor signalling. Consistently, whole-mount immunostaining of iWAT revealed significant remodeling of tyrosine hydroxylase-positive sympathetic neurons and CD31-positive vasculature in LPD-fed SPF but not GF mice (Fig. 2i**, Extended Data Fig. 12**). Specifically, following LPD feeding, sympathetic neurons and vasculature alike developed finer, denser networks, particularly in regions with a strong accumulation of UCP1-expressing cells. These results suggest that a LPD enhances sympathetic innervation, which may further promote the browning process.

Notably, administration of the β3 adrenergic receptor agonist CL316,243 to control diet-fed GF mice increased *Ucp1* expression to levels comparable to LPD-fed SPF mice (Fig. 3k). It also induced robust browning in *Nr1h4*^−*/−*^ and *Fgf21*^−*/−*^ mice (Fig. 3l), indicating that these mice possess functional beige precursor cells and that signalling mediated by microbiota, bile acids, and FGF21 all converge on β3 adrenergic receptor pathways to promote browning.

## Induction of browning by 20 mouse-derived microbes

To determine whether the impact of a LPD on iWAT browning is transmissible via the microbiota, we transplanted GF mice with small intestinal (SI) luminal contents or faeces from SPF mice fed a LPD for 6 weeks. Browning was not observed in any of the recipient mice without LPD feeding, regardless of whether they received SI or faecal microbiota. In contrast, all LPD-fed recipient mice exhibited beige fat biogenesis, though the magnitude varied between individuals (**Extended Data Fig. 13a**). These findings suggest that the observed LPD-mediated browning requires an ongoing interaction between diet and microbiota.

**Figure 4:**
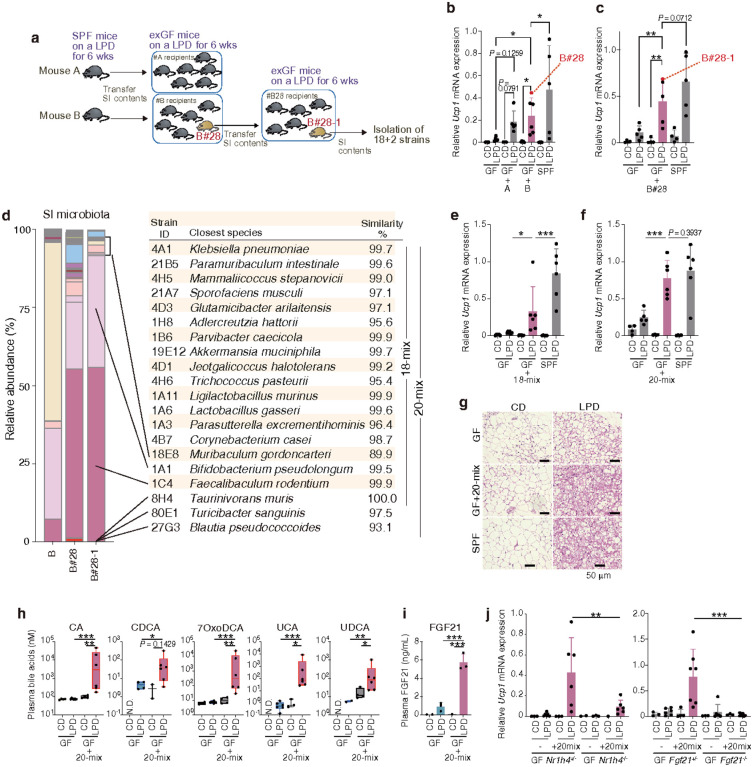
20 mouse-derived microbial isolates promote LPD-mediated browning. **a,** Schematic representing the strategy for isolation of browning-inducing commensal strains from the gut microbiota of SPF mice. **b, c, e, f,** GF mice were colonized with ileal microbiota from LPD-fed SPF mouse A, SPF mouse B, or exGF mouse B#28, or with defined bacterial consortia (18-mix or 20-mix). Gnotobiotic mice were fed a LPD for 6 weeks, and relative *Ucp1* expression in iWAT was measured by qPCR. **d,** Ileal microbiota compositions of mouse B, B#28, and B#28–1 were determined by 16S rRNA gene sequencing. The 20 strains isolated from mouse B#28–1 are listed. **g,** Representative H&E staining images of iWAT from the indicated groups of mice. **h, i,** Plasma bile acids **(h)** and FGF21 **(i)** were quantified in the indicated mice by LC-MS/MS and ELISA, respectively. **j,** *Ucp1* expression in iWAT from GF *Nr1h4*^−*/−*^ or GF *Fgf21*^−*/−*^ mice inoculated with 20-mix or vehicle control and fed a CD or LPD for 6 weeks. In **b, c, e, f, h-j,** each circle represents an individual animal and the height of each bar indicates the mean ± s.d. Horizontal lines in box plots indicate the median; box boundaries indicate the interquartile range; whiskers indicate the data range (g). ***P < 0.001; **P < 0.01; *P < 0.05; ns, not significant; analyzed by one-way ANOVA with Benjamini–Hochberg correction for multiple comparisons **(b, c, e, f, i, j)** or two-tailed Mann-Whitney test for each comparison **(h).** N.D., not detected.

To refine a bacterial community capable of promoting robust browning in the setting of low protein intake, we selected the mouse that exhibited the strongest *Ucp1* induction (mouse B#28) upon transfer of SI contents from LPD-fed SPF mice (denoted mice A and B) (Fig. 4a**, 4b, Extended Data Fig. 13b**). Subsequent administration of SI contents from mouse B#28 into new GF recipients recapitulated the efficient beige adipocyte biogenesis upon LPD feeding (Fig. 4a**, c, Extended Data Fig. 13c**). We selected mouse B#28–1 for follow-up analysis from among these recipients, as it exhibited the strongest beige cell induction (Fig. 4c). We then isolated 18 bacterial strains from the SI contents of mouse B#28–1 (Fig. 4d), colonized GF mice with a mixture of these strains (18-mix), and fed them a LPD. However, colonization with 18-mix was not sufficient to induce robust browning (Fig. 4e). We therefore isolated two additional strains from mouse B#28–1: strains St.27G3 and St.80E1 (Fig. 4d) [Note that the 16S rRNA gene sequences of St.27G3 and St.8031 are most similar to those in *Blautia pseudococcoide* and *Turicibacter sanguinis* but only share 93% and 97% similarity respectively, indicating that they represent previously undefined strains. For convenience, we refer to these isolates as *Blautia* sp. (St.27G3) and *Turicibacter* sp. (St.80E1) throughout this manuscript]. Colonizing GF mice with 18-mix plus these two additional strains (yielding 20-mix) and feeding a LPD led to robust *Ucp1* expression and accumulation of cells with beige morphology in the iWAT, to comparable levels as observed in SPF mice (Fig. 4f, g). These results suggest that the *Blautia* sp. (St.27G3) and *Turicibacter* sp. (St.80E1) strains play an essential role among the 20 strains. Importantly, GF+20-mix mice fed a LPD displayed elevated plasma levels of CA, CDCA, 7oxoDCA, UCA, and UDCA (Fig. 4h), as well as FGF21 (Fig. 4i). Notably, neither *Nr1h4*^−*/−*^ nor *Fgf21*^−*/−*^ GF mice colonized with 20-mix exhibited beige cell induction (Fig. 4j), confirming the critical roles of FXR and FGF21 signalling in LPD-induced, microbiota-mediated browning.

## Identification of 4 human-derived microbial isolates that promote browning

**Figure 5:**
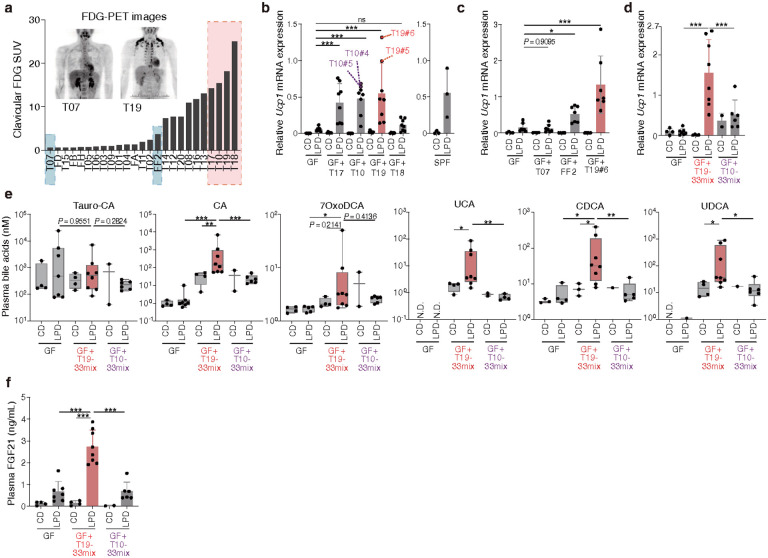
Down-selection of human-derived bacterial strains that induce LPD-mediated browning. **a,** Standardized uptake values (SUV) of FDG in the supraclavicular regions of each volunteer. Highlighted columns indicate samples that were selected for follow-up analysis, and representative FDG-PET images are shown as inserts. **b–f,** GF B6 mice were colonized with faecal microbiota from the indicated human subjects or with defined bacterial consortia and then fed either a CD or LPD for 6 weeks. Relative *Ucp1* expression in iWAT, normalized to *Ppib,* was determined by qPCR **(b-d).** Plasma bile acids **(e)** and FGF21 **(f)** were quantified by LC-MS/MS and ELISA, respectively. Each circle represents an individual animal. The height of each bar indicates exact SUV values **(a)** or the mean ± s.d. **(b-d, f).** Horizontal lines in box plots indicate the median; box boundaries indicate the interquartile range, and whiskers indicate the data range **(e).** ***P < 0.001 ; **P < 0.01; *P < 0.05; ns, not significant; analyzed by one-way ANOVA with Benjamini–Hochberg correction for multiple comparisons **(b-d, f)** or two-tailed Mann-Whitney test for each comparison **(e).** N.D., not detected.

Having thus confirmed that a small microbial community is sufficient to promote LPD-induced browning, we next sought to identify human-associated microbes capable of exerting this activity. To this end, we recruited 25 healthy volunteers and performed positron emission tomography (PET) using ^18^F-fluorodeoxyglucose (FDG), a tracer known to accumulate in brown and beige adipose tissue^17,79^. Approximately 40% of volunteers exhibited supraclavicular FDG accumulation (Fig. 5a). We transplanted faecal samples from the top 4 FDG-positive volunteers into GF mice and placed them on either a control or a low-protein diet (**Extended Data Fig. 14a**). exGF mice that received faecal transplants from donors T17, T10, or T19 and were fed a LPD displayed significant increases in beige adipocyte abundance and *Ucp1* expression in iWAT (Fig. 5b**, Extended Data Fig. 14b**). In contrast, faecal microbiota transplantation from donor T18 was far less effective, likely due to unsuccessful engraftment of key effector strains. We additionally transplanted faecal samples from donors FF2 and T07, who exhibited intermediate or no FDG accumulation, respectively. Mirroring with the human scenario, FF2 recipient mice exhibited an intermediate degree of browning, whereas T07 recipients showed no evidence of browning (Fig. 5c**, Extended Data Fig. 14c**).

To identify microbes capable of inducing browning, we isolated 33 strains from faecal samples from T10 microbiota-recipient mice (T10#4 and T10#5), and another 33 strains from the T19 microbiota-recipient group (mice T19#5 and T19#6) (Fig. 5b**, Extended Data Fig. 14d**). GF mice were colonized with each of these 33-strain mixtures. Mice colonized with the T19-derived 33-strain mixture exhibited robust beige cell induction upon LPD feeding, while those colonized with the T10-derived mixture showed substantially less (Fig. 5d**, Extended Data Fig. 14e**). Mice colonized with the T19-derived 33-mix exhibited increased plasma levels of CA, 7oxoCA, UCA, CDCA, and UDCA, as well as FGF21 (Fig. 5e, f), suggesting that these strains promote beige cell induction through activation of the FXR and FGF21 pathways.

**Figure 6:**
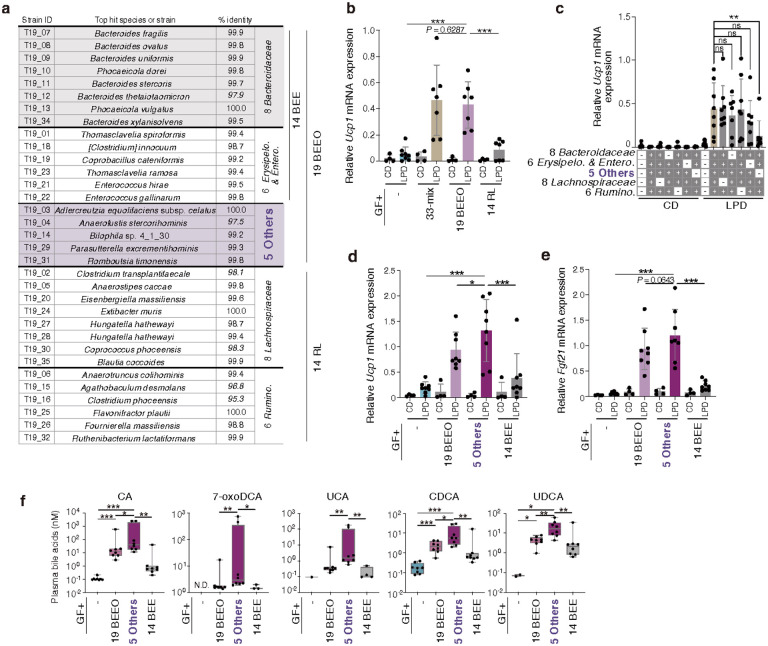
Identification of five human-derived isolates that promote browning. **a,** List of the 33 T19-derived strains, as determined by 16S rRNA sequencing. **b–f,** GF B6 mice were colonized with the indicated bacterial consortia and fed a CD or LPD for 4 weeks. Expression of *Ucp1* in iWAT **(b, c, d)** and *Fgf21* in the liver **(e),** normalized to *Ppib,* were measured by qPCR. Plasma bile acids in the indicated LPD-fed gnotobiotic mice were quantified by LC-MS/MS **(f).** Each circle represents an individual animal and the height of each bar indicates the mean ± s.d. **(b–e).** Horizontal lines in box plots **(f)** represent the median; box boundaries indicate the interquartile range; and whiskers indicate the data range. ***P < 0.001; **P < 0.01; *P < 0.05; ns, not significant; analyzed by one-way ANOVA with Benjamini–Hochberg correction for multiple comparisons **(b–e)** or two-tailed Mann-Whitney test for each comparison **(f).** N.D., not detected.

**Figure 7:**
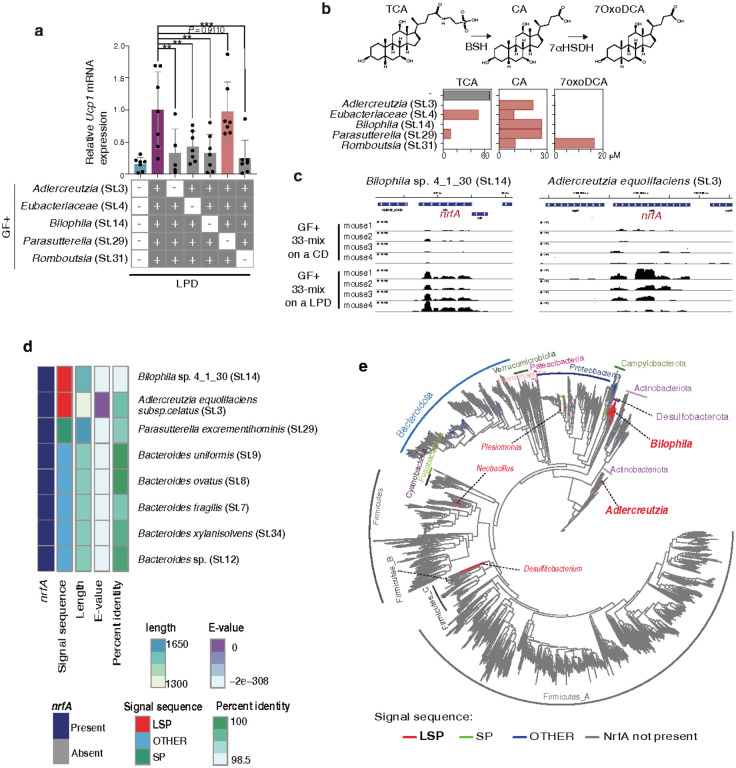
Exploring the role of four human-derived isolates in promoting browning **a,** GF B6 mice were colonized with the indicated bacterial consortium selected from 5 Others strains (‘+’ indicates inclusion and ‘−’indicates exclusion) and fed a LPD for 4 weeks. Relative *Ucp1* expression in iWAT, normalized to *Ppib,* was measured by qPCR. **b,** *In vitro* bile acid metabolic capacity of the 5 Others strains when starting with 50 μM taurine-conjugated cholic acid. Each circle represents an individual animal and the height of each bar indicates mean ± s.d. **(a)** or exact bile acid concentration **(b).** ***P < 0.001; **P < 0.01; *P < 0.05; ns, not significant; analyzed by one-way ANOVA with Benjamini–Hochberg correction for multiple comparisons **(a). c,**Metatranscriptomic analysis of bacterial RNA extracted from the caecal contents of gnotobiotic mice colonized with T19-derived 33-mix and fed a CD or LPD. Sequence reads are mapped to the *nrfA* genomic loci of *Bilophilasp*. 4_1_30 (St.14) and *Adlercreutzia equolifaciens* (St.3). **d**, Eggnog-mapper was used to annotate NrfA proteins. Signal sequences of *nrfA*homologues were analyzed using SignalP6.0. *Bilophila* sp. 4_1_30 (St.14) and *Adlercreutzia equolifaciens* (St.3) possess unique lipoprotein signal sequences (LSP), which are predicted to facilitate localization to the periplasm. “Percent identity” reflects homology to the closest NrfA protein, as assigned by Eggnog-mapper and calculated with BLASTP. “E-value” represents the expect value, indicating the number of chance alignments with an equal or higher bit score, as computed by BLASTP. “Length” indicates the length of the protein from each isolate that best aligns to NrfA as predicted by Eggnog-mapper. **e**. *nrfA* was annotated in all reference genomes from the Unified Human Gastrointestinal Genome (UHGG) using Eggnog, and signal sequences were analyzed using SignalP. “SP” stands for Sec/SPI: the “standard” secretory signal peptide putatively transported by the Sec translocon and cleaved by Signal Peptidase I, whereas LSP is Sec/SPII: lipoprotein signal peptide known to be transported by the Sec translocon and cleaved by Signal Peptidase II. Species carrying LSP containing *nrfA* homologues are highlighted in red.

To identify a minimal effector consortium, we divided the 33 T19-derived strains into 19 *Bacteroides, Enterococcus, Erysipelotrichaceae,* and other phyla strains (referred to as 19BEEO) and 14 *Ruminococcaceae* and *Lachnospiraceae* stains (referred to as 14RL) based on their phylogenetic relationships (Fig. 6a). 19BEEO-mix-colonized gnotobiotic mice recapitulated the robust iWAT *Ucp1* induction observed in those colonized with the parental 33-mix, whereas 14RL-mix-colonized mice did not (Fig. 6b). We further subdivided the 33 T19-derived strains into five phylogenetic groups and assessed the impact of excluding each on iWAT browning. Excluding the 5 strains classified as “Other” phyla led to the greatest reduction in *Ucp1* induction (Fig. 6c). We thus compared the effects of colonizing with the 5 Others-mix versus the remaining 14 strains from 19-mix, comprising *Bacteroides, Enterococcus,* and *Erysipelotrichaceae* species (referred to as 14BEE). Colonization with the 5 Others-mix efficiently induced beige cells in a LPD-dependent manner, whereas that with the 14BEE strains produced only a marginal effect (Fig. 6d). Colonization with the 5 Others-mix, but not 14BEE-mix, also led to elevated levels of both hepatic *Fgf21* mRNA transcripts and plasma bile acids, comparable to those observed in mice colonized with the parental 19BEEO-mix (Fig. 6e, f). Additional dropout experiments were conducted by inoculating GF mice with all possible 4-strain permutations of the 5 Others-mix, each omitting one of the 5 strains. Notably, omitting *Romboutsia timonensis* (St.31) prevented engraftment of the remaining 4 strains, thereby abrogating beige cell induction (Fig. 7a, **Extended Data Fig. 15a**), suggesting that *R. timonensis* acts as a supporter strain and facilitates the colonization by the others. Excluding either *Adlercreutzia equolifaciens* (St.3), *Eubacteriaceae* spp. (St.4), or *Bilophila* sp. 4_1_30 (St.14) did not affect colonization by the remaining strains but substantially impacted their ability to induce iWAT browning (Fig. 7a, **Extended Data Fig. 15a**). In contrast, omitting *Parasutterella excrementihominis* (St.29) had no impact on beige cell induction (Fig. 7a). Therefore, while the *Parasutterella* strain could be excluded, all 4 remaining strains (*Adlercreutzia, Eubacteriaceae, Bilophila’* and *Romboutsia*; collectively referred to as “hu4 strains”) are essential for the observed LPD-mediated browning effect.

## Role of *Bilophila* NrfA in browning

We next examined the bile acid-metabolizing capabilities of the 4 human-derived strains (St.3, St.4, St.14, and St.31). All hu4 strains exhibited bile salt hydrolase activity, converting taurocholic acid (tauro-CA) to CA *in vitro* (Fig. 7b). Furthermore, the supporter *R. timonensis* (St.31) strain, but not the others, carried a gene encoding a putative protein with a high homology (>60%) to 7α-hydroxysteroid dehydrogenase (HSDH) (**Extended Data Fig. 15b**). Consistently, this *R. timonensis* (St.31) strain was uniquely able to convert tauro-CA into 7oxoDCA (Fig. 7b). which may both enable colonization by the other strains and also activate FXR in iWAT.

**Figure 8:**
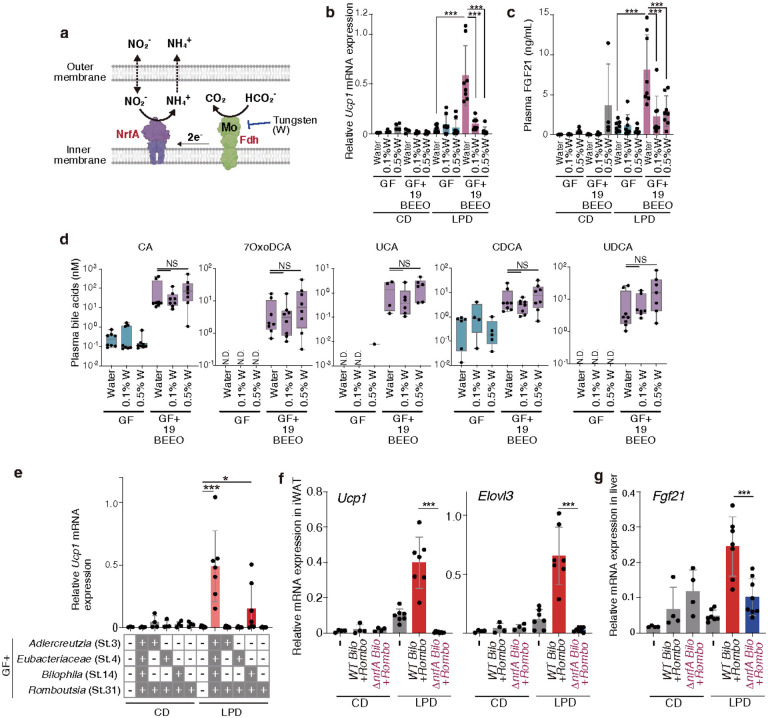
*Bilophila-*encoded *nrfA*contributes to LPD-mediated browning. **a,** Schematic illustrating nitrite reduction by NrfA in combination with formate dehydrogenase (Fdh). **b–d,** GF B6 mice were colonized with 19BEEO-mix and fed a CD or LPD for 3 weeks. During the last 2 weeks, mice were treated with 0.1% or 0.5% sodium tungstate dihydrate (W) via the drinking water. Relative *Ucp1* expression in iWAT **(b),** plasma FGF21 levels **(c),** and plasma bile acid concentrations **(d)** were measured using qPCR, ELISA, and LC-MS/MS, respectively. **e,** GF B6 mice were colonized with the indicated 2 bacterial strains selected from the hu4-mix (‘+’ indicates inclusion and ‘−’indicates exclusion) and fed a CD or LPD for 4 weeks. Relative *UcP1* expression in iWAT, normalized to *Ppib,* was measured by qPCR. **f, g,** GF B6 mice were colonized with either Δ*nrfAor* wild-type (WT) *Bilophila* sp. 4_1_30 (St.14) (*Bilo*) in combination with *Romboutsia timonensis* (St.31) (*Rombo*) and fed a CD or LPD for 4 weeks. Expression of *Ucp1* and *Elovl3* in iWAT and *Fgf21in* the liver, normalized to *Ppib,* were determined by qPCR. Each circle represents an individual animal and the height of each bar indicates the mean ± s.d. (**b, c, e-g**). Horizontal lines in box plots (**d**) represent the median; box boundaries indicate the interquartile range; and whiskers indicate the data range. ***P < 0.001; **P < 0.01; *P < 0.05; ns, not significant; analyzed by one-way ANOVA with Benjamini–Hochberg correction for multiple comparisons (**b, c, e–g**) or two-tailed Mann-Whitney test for each comparison (**d**). Mo, molybdopterin, N.D., not detected.

To further elucidate the mechanism by which these strains promote LPD-mediated browning, we sequenced the genomes of the all T19-derived 33 strains (**Supplementary Table 1**) and performed bacterial transcriptomic profiling on faecal samples from gnotobiotic mice colonized with this 33-mix and fed either a control or a low-protein diet. Enrichment analysis revealed that the genes most prominently upregulated upon LPD administration in both the 19BEEO and the hu4 strains, but not the 14BEE strains, were associated with nitrogen metabolism (**Extended Data Fig. 15c**). In particular, transcription of the gene encoding nitrite reduction by formate A (*nrfA*), which catalyzes reduction of nitrite into ammonia, was greatly increased in *Bilophila* sp. 4_1_30 (St.14) and *A. equolifaciens* (St.3) in LPD-fed mice (Fig. 7c). Although *nrfA* gene homologues are carried by various *Bacteroides* species, the *nrfA* gene sequences in our *Bilophila* and *Adlercreutzia* strains are unique in that they contain lipoprotein signal peptides (LSPs) (Fig. 7d). A search across all representative microbial genomes found in the human gut showed that 266 genomes across 9 phyla carry the *nrfA* gene. Of these, only 10.5% contain LSPs, and the vast majority of these LSP-*nrfA*-carrying species are in the phylum *Desulfobacterota,* of which *Bilophila* sp. 4_1_30 (St.14) is a member. Additionally, *A. equolifaciens* is the only *Actinobacteriota* with an LSP-containing NrfA (Fig. 7e**, Supplementary Table 2**). LSPs are predicted to facilitate transport via the Sec translocon, cleavage by signal peptidase II, and anchoring to the inner membrane^80^, thereby allowing NrfA to function in the periplasm, where it participates in the dissimilatory nitrate reduction to ammonium (DNRA) pathway. In this process, NrfA detoxifies nitrite and minimizes nitrogen loss by converting it into reusable, water-soluble ammonia instead of N_2_ gas^81,82^ (Fig. 8a). NrfA utilizes formate as an electron donor via its partner enzyme, formate dehydrogenase (Fdh)^81^. Fdh contains a molybdopterin cofactor in its active site, which can be inhibited by tungsten treatment^83^. Adding tungsten to the drinking water significantly suppressed both iWAT browning and plasma FGF21 levels in mice colonized with 19BEEO-mix (Fig. 8b, c), without impacting the colonization capacity of any of the strains including the 4 critical ones (**Extended Data Fig. 16c**). Importantly, tungsten treatment had no significant effect on plasma bile acid levels (Fig. 8d). Thus, although tungsten may additionally inhibit enzymes other than Fdh, these findings suggest that products of nitrogen metabolism play an important role in FGF21 production, and by extension, beige cell induction.

To investigate the contribution of NrfA to WAT browning, we generated a *nrfA-*deficient *Bilophila* sp. 4_1_30 (St.14). While both the wild-type and Δ *nrfA* strains were unable to monocolonize the mouse intestine, they engrafted successfully when co-administered with the supporter *R. timonensis* (St.31) strain (**Extended Data Fig. 17a**). Co-colonization with wild-type *Bilophila* sp. 4_1_30 (St.14) and *R. timonensis* (St.31) induced significant browning, though the magnitude was substantially less than that observed with the complete hu4 strains (Fig. 8e). *Bilophila* sp. 4_1_30-mediated browning was completely dependent on NrfA competence, as *R. timonensis* co-colonization with the Δ *nrfA* mutant failed to induce beige cells (Fig. 8f). Notably, *nrfA* deficiency also suppressed hepatic *Fgf21* expression (Fig. 8g), but did not affect *Csad* and *Cyp39a1* expression (**Extended Data Fig. 17b**). These results suggest that NrfA-mediated nitrogen metabolism promotes the induction of WAT browning via upregulation of FGF21. Collectively, our data suggest that LPDs modulate the function of specific microbiota members, which in turn activate several pathways to exert wide-reaching influence over multiple organ systems, ultimately promoting the induction of beige cells.

## Discussion

In this study, we demonstrate the effectiveness of dietary modulation in altering microbiota function and inducing WAT browning, thereby potentially influencing host metabolism. LPD-mediated browning is not entirely dependent on the pathways activated by traditional browning stimuli such as cold exposure. Whereas the immune system plays a pivotal role in the induction and activation of beige cells in response to cold exposure or other stimuli, such as high fiber diets (Mohammad Arifuzzaman and David Artis, personal communication), LPD-mediated browning is largely independent of the immune system but depends heavily on specific members of the gut microbiota. Previous reports have shown that the gut microbiota can both positively and negatively influence WAT browning, with some studies linking it to enhanced adaptive thermogenesis and others implicating it in undesirable metabolic processes like inhibition of browning and promotion of insulin resistance and glucose intolerance^33^. By isolating and transplanting specific bacterial strains from mice or humans into GF mice, we have successfully demonstrated that certain members of the gut microbiota can sense low protein conditions and strongly promote WAT browning. In particular, 4 human-derived isolates (namely *Adlercreutzia, Eubacteriaceae, Bilophila,* and *Romboutsia* strains) were found to play a critical role in promoting browning in response to a LPD. These specific bacteria can increase the concentration of certain bile acids in the systemic circulation, which presumably travel to and activate FXR expressed by adipose progenitor cells. Additionally, these bacteria stimulate the liver to produce FGF21, likely by sensing nitrogen scarcity and activating the compensatory DNRA program. Since FGF21 deficiency did not affect bile acid levels, and FXR deficiency did not alter FGF21 levels, these pathways likely operate in parallel, each ostensibly contributing to the differentiation and accumulation of beige adipocytes (**Extended Data Fig. 18**). Furthermore, the interaction between low protein intake and the microbiota leads to dramatic changes in sympathetic neurons in WAT. Therefore, consistent with previous reports^50,84^, FXR and FGF21 receptor signaling extends beyond simply promoting adipocyte differentiation and affects various cell types in WAT. Of course, further studies are needed to clarify how the microbiota senses low-protein conditions, how the LPD-conditioned microbiota preferentially increases certain bile acids in the systemic circulation, how specific microbiota members exert their influence across disparate organs, and how FGF21, FXR, and possibly other factors lead to a dramatic remodeling of sympathetic neurons. Nevertheless, our results, along with previous reports^8–12,15,28,30,31,53,70–75,78,^ highlight the complexity of pathways involved in browning and open numerous avenues for future research and therapeutic translation.

Our data shows that a LPD ameliorated metabolic conditions such as obesity and fatty liver disease in both mice and humans. Indeed, pharmacological activation of FXR and FGF21 receptors has been demonstrated to enhance thermogenesis and WAT browning in animal models, and several FXR modulators and FGF21 analogues are currently under clinical evaluation for the treatment of metabolic diseases in humans^85,86^. A LPD can activate both pathways simultaneously, making it a promising therapeutic avenue while skirting challenges related to clinical trials involving chemical compounds and biologics. A reduction in dietary protein content is widely used to treat patients with chronic kidney disease. Moreover, vegan and vegetarian diets are becoming increasingly popular, paving the way for easier integration of moderate protein restriction in metabolic disease management. Recently, high-protein and ketogenic diets have gained popularity for maintaining body weight and health, despite controversies regarding their efficacy. Our data suggest that the effects of dietary modulation may be context-dependent. By combining live biotherapeutic products functionally equivalent to those identified in this study, the effects of a LPD are likely to become more specific and potent. Further exploring the interactions between different strains and their collective impact on host physiology will be crucial for optimizing microbiota-based therapies.

Despite the significant findings, our study has several limitations. While our mouse models provide valuable insights, translating these results to humans requires careful consideration. Our preliminary clinical trial assessing the efficacy of commercially available LPDs for treating patients with fatty liver disease, though promising, was limited by a small sample size and a short intervention period. Larger, long-term studies are needed to validate the efficacy and safety of LPDs in humans, particularly with regards to the potential risk of inducing frailty, especially in aged individuals. Additionally, it remains unclear whether LPDs promote beige cell accumulation in humans. Moreover, genetic and environmental variability among individuals may influence the effectiveness of dietary interventions and microbiota-based therapies, and thus personalized approaches that account for individual microbiota compositions and dietary habits may be essential to achieve optimal outcomes. Future human studies must address these challenges. Nevertheless, our study provides important insights into the role of the gut microbiota in promoting LPD-mediated WAT browning and lays the groundwork for developing and translating novel dietary and microbiota-based interventions to combat prevalent metabolic diseases.

## Methods

### Mice

Specific pathogen-free C57BL/6 (B6), BALB/c and ICR mice were purchased from Japan SLC, CLEA Japan and Jackson Laboratory Japan. Male B6 mice aged 7 to 17 weeks were used unless otherwise specified. Germ-free (GF) male B6 mice were purchased from Sankyo Labo Service Corporation and CLEA Japan. GF rederivation of SPF mutant mice was conducted at the gnotobiotic facilities of RIKEN and Keio University. Briefly, *in vitro* fertilization (IVF) was used to generate embryos (typically using eggs and sperm from heterozygous pairs), which were then transplanted into IQI pseudo-pregnant female recipients. Following embryo transfer, the recipient females underwent Caesarean section on embryonic day 18. The intact uterine horns containing the pups were passed through a germicidal bath, after which the pups were delivered into a flexible plastic GF isolator and suckled by a GF lactating foster mother. This method enabled the generation of GF cohorts of ten to forty mice, all born on the same date (littermates or equivalents), thereby supporting consistent experimental conditions. *Fgf21*^*−*/−^ mice were generated as previously described^87^. Triple knockout mice lacking β1-, β2-, and β3-adrenergic receptors (*Adrb1*^*−*/−^*, Adrb2*^*−*/−^*, Adrb3*^*−*/−^) were utilized with permission from Dr. Bradford Lowell from Beth Israel Deaconess Medical Center^88^. *II4*^*−*/−^ mice (G4 mice) were kindly supplied by Dr. William E. Paul of the National Institute of Allergy and Infectious Diseases^89^. R26:lacZbpA^fl^°^x^DTA mice were provided by Dr. Dieter Riethmacher from Nazarbayev University School of Medicine 90 and subsequently crossed with *Il5-*Cre or *LysM*-Cre mice. *Nr1h4*^−*/−*^*, Gpbar1*^*−/−*^*, Nrlh4*^fl/fl^*, Villin-Cre, Albmin-Cre, Adipoq-Cre, Tcrb*^*−/−*^*, Tcrd*^*−/−*^*, Tbx21*^*−/−*^*, Rorc*^*−/−*^ (homozygous of Rorc(γt)-EGFP mice), *Il5-Cre* and *Lta*^*−/−*^ micewere purchased from Jackson Laboratories. *Rag2*^*−/−*^*γc*^*−/−*^ mice was obtained from Taconic. *II33*^*−/−*^ mice were obtained from RIKEN BRC with permission from Dr. Susumu Nakae (Oboki et al., 2010). All mice were housed under controlled conditions, including a temperature range of 23–25°C, regulated humidity, and a 12-hour light/dark cycle. Mice were provided with water and gamma-irradiated food *ad libitum* throughout the experiments. The types of food and the duration of the experiments are specified in each figure panel. All animal experiments were approved by the Keio University Institutional Animal Care and Use Committee and the RIKEN Yokohama Institute.

### Experimental diets

All experimental diets were obtained from Research Diets or Oriental Yeast. The control diet (CD) used in this study was a 50 Gy-irradiated AIN-93G diet (product #D19090404) containing 20 kcal% protein (mineral acid casein), 64 kcal% carbohydrate, and 16 kcal% fat. Unless otherwise specified, the low-protein diet (LPD) was an isocaloric AIN-93G-based diet containing 7 kcal% protein, 77 kcal% carbohydrate, and 16 kcal% fat (product #D20121501; see **Supplemental Table 3,4**). Diets with varying proportions of protein, carbohydrates, and fats were formulated based on the AIN-93G diet, with the exception of the Ketogenic diet (KD) and the KD-control diet, which contain lower fat content compared to AIN-93G. The KD and KD-control diets (product #D20012303 and #D20012304 from Research Diets) use cocoa butter as the primary fat source, with protein concentration adjusted to 20 kcal% to match the AIN-93G diet. Because the KD-control diet contains only 10% fat, which is lower than that of AIN-93G, it is referred to as the “Low fat” diet in Figure 1a. For experiments using defined amino acid diets, natural protein (mineral acid casein) was replaced with pure amino acids, aligned with the amino acid profile of the protein. The total amino acid concentration was then adjusted, ranging from 20 to 2.5 kcal%, or set to 2.5% kcal content for individual essential amino acids, while maintaining the levels of other amino acids at 20% and total calorie was compensated with varying the amount of carbohydrate to be isocaloric (see **Supplemental Table 5**). The high-fat diet was purchased from CLEA Japan (product #HFD32). The GAN (Gubra Amylin NASH) diet, containing 40 kcal% fat, 20 kcal% fructose, and 2 g% cholesterol (product #D09100310N from Research Diets), was used as the MASH (metabolic dysfunction-associated steatohepatitis) diet.

### Cold exposure, β3-adrenergic receptor agonist, or tungsten treatment

For cold exposure, mice were housed at 6°C for 7 days. For treatment with a β3-adrenergic receptor agonist, mice were administered daily intraperitoneal injection of CL316,243 (20 μg/mouse/dose, SIGMA) for 7 consecutive days. For tungsten treatment, mice were provided with 0.22 μm-filter-sterilized sodium tungstate dihydrate (Na WO 2H •O, Nacalai Tesque #32011–25) in their drinking water at concentrations of 0.1% or 0.5% for 2 weeks. During the treatment period, animals had unrestricted access to the tungsten solution, and the remaining volume and the animal health was carefully monitored.

### Quantitative Reverse Transcription Polymerase Chain Reaction (qPCR)

To evaluate mRNA expression, whole iWAT (excluding inguinal lymph nodes), gWAT, BAT, and 30–50 mg of liver and ileum tissue were homogenized using 1.4 mm ceramic beads. Total RNA was extracted using TRIzol reagent (Invitrogen) following the manufacturer’s protocol. For iWAT and gWAT lysates, the lipid fraction was removed at the initial step by centrifugation. For qPCR analysis, cDNA was synthesized from 0.5 μg of total RNA using ReverTra Ace qPCR RT Master Mix (TOYOBO), and qPCR was performed with Thunderbird SYBR qPCR Mix (TOYOBO) on a LightCycler 480 II (Roche). The following primer pairs were used: *Ppib,* 5’-GGAGATGGCACAGGAGGAA-3’ and 5’-GCCCGTAGTGCTTCAGCTT-3’; *Ucp1,* 5’- CACCTTCCCGCTGGACACT -3’ and 5’-CCCTAGGACACCTTTATACCTAATGG-3’; *Elovl3,* 5’-tgttggccagacctacatga-3’ and 5’-ggcccactgtaaacatcactg-3’; Cidea, 5’-ATCACAACTGGCCTGGTTACG-3’ and 5’-TACTACCCGGTGTCCATTTCT-3’; *Cox7a1,* 5’-AGCTGCTGAGGACGCAAAAT-3’ and 5’-CTTCTCTGCCACACGGTTTT-3’; *Cox8b,* 5’- GAACCATGAAGCCAACGACT-3’ and 5’-GCGAAGTTCACAGTGGTTCC-3’; Eno1, 5’-TGCGTCCACTGGCATCTAC-3’ and 5’-CAGAGCAGGCGCAATAGTTTTA-3’; *Ldhb,* 5’-TGTCTCCAGCAAAGACTACTGT-3’ and 5’-GACTGTACTTGACAATGTTGGGA-3; *Pdha,* 5’-GAAATGTGACCTTCATCGGCT-3’ and 5’-TGATCCGCCTTTAGCTCCATC-3; *Phgdh,* 5’-ATGGCCTTCGCAAATCTGC-3’ and 5’-AGTTCAGCTATCAGCTCCTCC-3’; *Psat1,* 5’-TGCCACACTCGGTATTGTTG-3’ and 5’-CAGCTAGCAATTCCCTCACAA-3’; *Aldh1l2,* 5’-AAAGAGGGCCACCGAGTAGT-3’ and 5’-TTCATCGAGGGAACTTGAAC-3’; *Asns,* 5’-AAGATGGGTTTCTGGCTGTG-3’ and 5’-ACAGACGCAACTTTGCCATT-3’; *Gdf15,* 5’-GAACCAAGTCCTGACCCAGC-3’ and 5’-GCTTCAGGGGCCTAGTGATG-3’; Fgf21, 5’-CCTGGGTGTCAAAGCCTCTA-3’ and 5’-TCCTCCAGCAGCAGTTCTCT-3’; *Cyp39a1,* 5’-TTCTCACCAATAGCAATCGCC-3’ and 5’-GTCATTCGGTTTCCCATAGCAA-3’; *Csad,* 5’-GCCGGACTGTGATTCACTACA-3’ and 5’-GTGTTGAGGCTCTCCGTGAT-3’. In most experiments, Ct values were converted into quantities using linear equations derived from standard curves, which were generated from serial dilutions of cDNA samples containing high copy numbers of the target gene. In cases where no samples with high copy numbers or obvious positive controls were available, the ΔΔCt method was used. Ppib (peptidylprolyl isomerase B) served as the housekeeping gene for normalization, enabling the calculation of relative expression levels for each target gene.

### Bulk RNAseq analysis of iWAT and liver

Libraries were prepared using the TruSeq Stranded mRNA Library Prep Kit (Illumina) following the manufacturer’s instructions. Sequencing was performed by Macrogen Japan Corp. using a NovaSeq 6000 platform (Illumina) with 100-bp paired-end reads and a run scale of 4 Gb per sample. Sequenced reads were mapped to the mouse reference genome (mm10) and normalized to reads per kilobase per million reads (RPKM) using Strand NGS software v.2.7 (Strand Life Sciences). The summarized data were then assessed by statistical models (one-way ANOVA with TukeyHSD and Benjamini–Hochberg for multiple gene correction).

### Metabolic cage analysis

Oxygen consumption (VO) and carbon dioxide production (VCO) were measured every 3 minutes over a 2-hour period during the day (12:00 pm–2:00 pm) and night (12:00 am–2:00 am) using an open-circuit metabolic gas analysis system connected to a mass spectrometer (Arco2000; ArcoSystem). The respiratory quotient was calculated as the ratio of VCO to VO. Locomotor activity and food intake were continuously monitored for 5 days using an activity monitoring system (ACTIMO-100; Shinfactory) integrated into individual metabolic chambers. For the oral glucose tolerance test (OGTT), mice were fasted overnight and then administered glucose orally at a dose of 1.2 g/kg. Blood samples were taken from the tail before and at 15, 30, 60, 90, and 120 minutes after glucose administration. Blood glucose levels were measured using GlucoCard G Black sensors and blood glucose test strips (G sensor, Arkray).

### Histological analysis

Freshly harvested tissues were fixed in 4% paraformaldehyde, embedded in paraffin, and sectioned into 5-μm slices before undergoing hematoxylin and eosin (H&E) staining. For immunostaining, tissues were first deparaffinized three times in xylene and then rehydrated. The sections were blocked for 60 minutes in PBS containing 5% goat serum, 1% BSA, and 0.5% Tween20. After rinsing in PBS, the slides were incubated overnight at 4°C with Alexa Fluor647-conjugated anti-UCP1 antibody (rabbit, EPR20381, Abcam #ab225489, 1:200). Following additional washes, the sections were stained with DAPI, mounted, and imaged using a BZ-X810 microscope (Keyence).

### Whole-mount immunostaining

After the collection of iWAT tissues, they were postfixed overnight in 4% paraformaldehyde (PFA) and stored in 80% ethanol at 4°C until iDISCO processing. Immunohistochemical staining was performed following the detailed experimental protocol of iDISCO from Huesing, C. et al. ^91^ with modifications. iWAT samples were dehydrated in a series of 1-hr methanol (MeOH)/H_2_O concentrations (20, 40, 60, 80, and 100%), then incubated overnight at room-temperature in a 66% dichloromethane (DCM) and 33% MeOH solution on a rocker (multi bio 3D programmable mini-shaker from Biosan company). Tissues were washed twice in 100% methanol and were treated with 1:5 concentration solution of 30% H_2_O_2_: 100% MeOH overnight at 4°C. Following overnight incubation, tissues were rehydrated in a series of 1-hr MeOH/H2O washes, then washed in fresh PBS overnight on a rocker at room temperature. Tissues were washed with PTx.2 solution (0.2% TritonX-100 in PBS) twice on a rocker at 1-hr intervals. Samples were then incubated in permeabilization solution (400mL PTx.2, 11.5g glycine, 100mL dimethylsylfoxid (DMSO)) for 2 days at 37°C in a shaker (Bio Shaker, BR-43FL). This was followed by the incubation in blocking solution [42 mL PTx.2, 5 mL DMSO, 3 mL normal donkey serum (NDS)] at 37°C for 2 days in the shaker. Then tissues were incubated with primary antibodies [chicken anti-TH (1:400, Milipore, Cat-AB9702); rabbit anti-UCP1 (1:400, Abcam, ab225489); goat anti-CD31 (1:300, Novus Biologicals, AF3628)] in primary incubation solution [92 mL PTwH (1 mL of 10 mg/mL heparin stock solution, 2 mL Tween-20 dissolved in 1 liter of PBS), 5 mL DMSO, 3 mL NDS] on a shaking incubator at 25°C for 7 days. Following primary antibody staining, tissues underwent six, 1-hr washes in PTwH then subjected to secondary antibody staining [AlexaFluor555 donkey anti-chicken (1/300, ThermoFisher Scientific, A-78949); AlexaFluor488 donkey anti-GoatIgG (1:300, ThermoFisher Scientific, A-11055)] in secondary incubation solution (PTwH with 3% NDS) for 10 days at 25°C on a shaker. Samples underwent the same PTwH wash step and incubated overnight at room temperature on a rocker with fresh PTwH solution. Additional round of MeOH dehydration was performed (described above) and incubated overnight in 100% MeOH on a rocker at room temperature. Tissues were submerged in 66%DCM/33%MeOH solution for 3 hr. Once completed, the samples underwent two, 100% DCM washes, and were then placed in dibenzyl ether (DBE), an organic solvent, for 3 days on a rocker at room temperature for clearing. A ZEISS Lightsheet 7 microscope or Miltenyi Biotec UltraMicroscope Blaze were used to generate three-dimensional (3D) images of iWAT tissues with a zoom factor of 5x or 20x. Images of TH and UCP1 labelling were collected for all samples using ZEN black, ZEN blue and Arivis software.

### Blood FGF21 measurement

Plasma samples were collected from mice via cardiac puncture under proper anesthesia and stored at −80°C until analysis. FGF21 was measured using mouse FGF-21 DuoSet ELISA (R&D) according to the manufacturer’s instructions.

### Western blot analysis

Mouse iWAT was snap-frozen in liquid nitrogen and the proteins were extracted using RIPA buffer, and the final protein concentration was adjusted to 4 μg μl-1. For SDS–PAGE and blotting, the Novex NuPAGE SDS–PAGE Gel system (Thermo Fisher Scientific) and Trans-Blot Turbo system (Bio-Rad, 1704150) were used according to the manufacturer’s instructions. iBind Western Systems (Thermo Fisher Scientific) were used for staining throughout the study. The antibodies used in this study are as follows: rabbit anti-mouse UCP1 (Abcam, ab10983, 1:1000), mouse monoclonal anti-b-actin antibody (Sigma, A1978, 1:1000), goat anti-rabbit IgG HRP-linked (Cell signaling, #7074, 1:2000), horse anti-mouse IgG, HRP-linked (Cell signaling, #7076, 1:2000). Chemi-Lumi One (nacalai tesque) was used for the chemiluminescence assays and the Fusion FX (Vilber) was used for imaging.

### FXR reporter assay

The FXR stimulating activity of bile acids was assessed using the GeneBLAzer FXR-UAS-bla HEK 293T Cell Agonist Assay (Thermo Scientific), following the manufacturer’s protocol. Briefly, cells were seeded in black-walled, clear-bottomed 384-well assay plates at a density of 1×10^4^ cells per well and incubated overnight at 37°C in a 5% CO2 atmosphere. Each of bile acids (CDCA, UDCA, CA, 7oxoCA, UCA, DCA, Tauro-βMCA, or Tauro-CA, all sourced from Cayman) were added at concentrations ranging from 20 to 0.61 μM. Following overnight incubation at 37°C, cells were treated with the FRET Blue/Green-enabled substrate CCF4-AM and incubated for an additional two hours at room temperature in the dark.

Fluorescence was measured using a TECAN microplate reader at emission wavelengths of 460 nm (Blue) and 530 nm (Green), with an excitation wavelength of 409 nm. The Blue/Green emission ratio for each well was calculated by dividing the background-subtracted blue emission values by the green emission values, as per the manufacturer’s instructions. Data from cells exhibiting abnormal morphology, indicative of cellular toxicity at higher bile acid concentrations, were excluded from the analysis.

### Single nucleus RNAseq analysis of iWAT

Approximately 50 mg of mouse iWAT around the inguinal lymph nodes was dissected, then the lymph nodes were removed, cut into about 20 pieces and stored in liquid nitrogen until use. Nuclei were isolated from the frozen samples using 10x Chromium Nuclei Isolation kit according to manufacturer’s protocol. An aliquot of nuclei from each sample was stained with trypan blue or acridine orange/propidium iodide (Logos Biosystems), counted in a haemocytometer and LUNA-FX7 to identify intact nuclei, and nuclei from 3 or 4 mice were pooled and immediately loaded on the 10x Chromium controller (10X Genomics) according to the manufacturer’s protocol. For each sample (GF+CD, GF+LPD, SPF+CD, SPF+LPD, n=2 pools, 8 samples in total), 20,000 nuclei were loaded in one channel of a Chromium Chip (10x Genomics). The Chromium Next GEM Single Cell 3^’^ v3.1 chemistry (Dual index) was used to process all samples. cDNA and gene expression libraries were generated according to the manufacturer’s instructions. cDNA and gene expression library fragment sizes were assessed with a DNA High Sensitivity Screen tape (Agilent). Gene expression libraries were multiplexed and sequenced on the NovaSeq6000 (Illumina) with the mode of 150-bp paired-end at Macrogen Japan. Cell Ranger v7.0 pipelines from 10x Genomics was used to align reads to the mm10 genome assembly and produce feature matrices. In order to adjust for downstream effects of ambient RNA expression within mouse nuclei, we used CellBender version 0.1.0^92^ to remove counts due to ambient RNA molecules from the count matrices and estimate the true nuclei. Seurat v4.3.0 (ref. ^93^) was used for QC, analysis of individual feature matrices and integrated analysis of all 8 samples (dim=8, resolution=0.5 for liver, dim=30, resolution=1.2 for iWAT) and the UMAP plot.

### Trajectory inference analysis

To study differentiation within the adipose cell subsets, we performed trajectory analysis using Slingshot^94^. We first subset our Seurat object to include only mature iWAT adipocyte clusters in SPF LPD-treated cells (Adipocyte10, Adipocyte03, Adipocyte07, Adipocyte05, Adipocyte09, Adipocyte02, Beige adipocyte, Adipocyte12, Adipocyte11, Adipocyte01, Adipocyte06, Adipocyte08, Adipocyte04). After subsetting, we converted the Seurat object to a SingleCellExperiment object (v1.26.0) using the as.SingleCellExperiment function and computed pseudotime lineages using Slingshot (v2.12.0) on the UMAP embeddings^95^. We next computed a cell-cell transition matrix and identified lineage driver genes using CellRank^96^. The Seurat object, along with Slingshot pseudotime coordinates, was converted to an AnnData object via Seurat’s Convert function for analysis in Python. Of the four lineages identified by Slingshot, we selected cells belonging to the Beige Adipocyte lineage (lineage 2). Within this lineage, we computed cell-cell transition probabilities using the PseudotimeKernel in CellRank (v2.0.5), which incorporates both a k-nearest neighbors (k-NN) graph (30 principal components and 50 nearest neighbors) and the Slingshot pseudotime ordering. We used the resulting cell-cell transition matrix to estimate fate probabilities and identify key driver genes within the beige cell lineage. We fit the Generalized Perron Cluster Cluster Analysis (GPCCA) estimator on the pseudotime kernel, assigning Beige adipocyte as the terminal state. We finally computed beige adipocyte fate probabilities and identified genes correlated with these fate probabilities using the compute_lineage_drivers function, with default parameter settings.

To further study these lineage driver genes, we visualized their expression along the beige cell lineage and performed pathway enrichment analysis. We visualized gene expression trends by fitting the expression of lineage driver genes along pseudotime using Generalized Additive Models (GAM).

### Single nucleus RNAseq analysis on liver

Nuclei were collected from the frozen liver tissues (approximately 50–60 mg) using Singulator 100 (S2 Genomics, with the inbuilt program ‘Single-Shot Standard Nuclei Isolation V2’.) and buffers according to protocol of 10x genomics (Demonstrated Protocol – Nuclei Isolation for Single Cell Multiome ATAC + GEX Sequencing). Resulting suspensions including nuclei were thorough 30mM strainer and stained with acridine orange/propidium iodide (Logos Biosystems), counted in a LUNA-FX7 to identify intact nuclei. Nuclei were immediately loaded on the 10x Chromium controller (10x Genomics) according to the manufacturer’s protocol. For each sample (GF+CD, GF+LPD, SPF+CD, SPF+LPD, n=2 mice, 8 samples in total), 8,000 nuclei were loaded in one channel of a Chromium Chip (10x Genomics). The Chromium Next GEM Single Cell 3^’^ v3.1 chemistry (Single index) was used to process all samples.

### Examination of the effects of LPD on patients with MAFLD

Adult outpatients with metabolic-associated fatty liver disease (MAFLD) who attended Jichi Medical University Hospital were recruited for this study if they met the inclusion criteria and provided informed consent. The inclusion criteria were: (1) evidence of liver injury due to MAFLD (ALT ≥ 40 U/L), (2) BMI ≥ 25, and (3) a controlled attenuation parameter (CAP) value ≥ 248 dB/m, as measured by FibroScan. Exclusion criteria included: sarcopenia, liver cirrhosis, alcoholic liver disease, autoimmune hepatitis, primary biliary cholangitis, viral hepatitis, Wilson’s disease, any liver disease other than MAFLD, uncontrolled or severe heart failure, diabetes, hypertension, interstitial pneumonia, renal failure, autoimmune diseases, infections, malignancies, insulin therapy, steroid therapy, dialysis, and pregnancy. After obtaining informed consent, registered dietitians provided dietary counseling and supplied commercially available LPD meals (https://wellness.nichirei.co.jp/shop/goods/search.aspx) to achieve a target protein intake of 0.6–0.8 g/kg of standard body weight per day. Participants followed the diet for two weeks. Dietary adherence and intake were monitored through meal photographs and dietary interviews, with dietitians calculating caloric, protein, carbohydrate, and fat intake. Body measurements, blood samples, and stool samples were collected before and after the dietary intervention. This trial is registered with the Japan Registry of Clinical Trials (jRCT1030210391) (https://jrct.niph.go.jp/latest-detail/jRCT1030210391). The study protocol adhered to the ethical standards of institutional and national committees on human experimentation, as well as the Declaration of Helsinki (1964 and later amendments). It was approved by the Institutional Review Board of Jichi Medical University (Rin21–076).

### FDG-PET scan

Twenty-five healthy male volunteers (age: 20 to 47 yo) were recruited to investigate the role of gut microbiota in beige cell accumulation. All participants were thoroughly briefed on the study and provided written informed consent. The protocols were approved by the Institutional Research Ethics Review Board of Tenshi College (Sapporo, Japan) (UMIN000016361). Human brown and beige cell activity was assessed using a FDG-PET scan (Aquiduo; Toshiba Medical Systems) following standardized non-shivering cold exposure, as described previously^79,97^. All subjects fasted for 12 hours before undergoing the PET-CT scan. After 1 hour of cold exposure, volunteers received an intravenous injection of ^18^F-FDG (1.66–5.18 MBq per kg body weight) and remained in the cold room for an additional hour. Brown and beige cell activity was evaluated by measuring the standardized uptake value (SUV) of ^18^F-FDG and Hounsfield units from −300 to −10 in the supraclavicular region using Fusion software (Toshiba Medical Systems). Fecal samples were collected from all participants and stored at Keio University following the protocol approved by the institutional review boards (approval number 20150075).

### Bacterial isolation and generation of gnotobiotic animals

Human and mouse faecal samples and intestinal contents were suspended in an equal volume (w/v) of PBS containing 20% glycerol, snap-frozen in liquid nitrogen, and stored at −80°C until use. To introduce into GF mice, the frozen stocks were thawed, suspended in mGAM broth, filtered through a 100 μm cell strainer, and an aliquot (approximately 2–5 mg in 250 μL per mouse) was orally inoculated into GF mice. To isolate beige cell-inducing bacterial strains associated with mice or humans, small intestinal contents from the B#28–1 mouse or faecal samples from mice colonized with T10 or T19 human microbiota were serially diluted in PBS and plated onto nonselective and selective agar plates. EG, BHK, and BBE media were used for the isolation of human-derived strains, while BL, Mucin medium, YCFA-GSC, Marine, and TSA were additionally used for the isolation of mouse-derived strains. After incubation under anaerobic conditions (80% N, 10% H, 10% CO) in an anaerobic chamber (Coy Laboratory Products) or under aerobic conditions at 37°C for 2 to 7 days, individual colonies were picked. The full-length 16S rRNA gene region was amplified using universal primers (27Fmod: 5’-AGRGTTTGATYMTGGCTCAG-3’, 1492R: 5’-GGYTACCTTGTTACGACTT-3’) and sequenced by sanger sequencing. The resulting strain sequences corresponding to the first half (approximately 0.8 kbp) were aligned and compared using BLAST to identify closely related species or strains. Individual isolates were classified as a “strain” if their 16S rRNA gene sequences exhibited 100% identity. The sequences were also compared to amplicon sequence variants (ASVs) identified in small intestinal samples from mouse B#28–1 and fecal samples from mice T10#4, T10#5, T19#5, and T19#6 to identify their corresponding ASVs. To prepare bacterial mixtures for inoculation into GF mice, individual strains were cultured to confluence in mGAM broth or BHKRS agar, and equal volumes of the resulting bacterial suspensions were mixed. Specific supplements were added to support the growth of certain strains: 0.1% fumarate, 0.1% formate, and 0.5 μg/mL vitamin K were added for *Parasutterellaceae* (St.1A3); 0.1% fumarate, 0.1% formate, 0.5 μg/mL vitamin K, and 0.1 mg/mL sodium sulfate were added for *Blautia* sp. (St.27G3), *Eggerthellaceae* (St.1H8), and *Taurinovorans muris* (8H4); and 1 mg/mL glycine and 1 mg/mL glutamate were added for *Parvibacter caecicola* (St.1B6). For *Bilophila* sp. 4_1_30 (St.14) or *Ruthenibacterium lactatiformans* (St.32), cultures were supplemented with 0.1% fumarate, 0.1% formate, 0.5 μg/mL vitamin K, 0.1 mg/mL sodium sulfate, 1 mg/mL glycine, and 1 mg/mL glutamate. In some experiments, 1% taurine was added for *Bilophila* sp. 4_1_30 (St.14), or 1% arginine was added for *Adlercreutzia* (St.3) strains. The bacterial mixtures were orally administered to GF mice, delivering approximately 1–10 × 10^8^ CFU of each strain in 250 μL of medium per mouse. All mice receiving the same mixture of bacterial strains were housed together in a single gnotobiotic isolator. Gnotobiotic mice were analyzed 4 or 6 weeks after the inoculation unless otherwise indicated. Colonization was evaluated through direct smears of faecal suspensions and qPCR analysis of fecal/cecal DNA, using the extraction method described in the 16S rRNA gene amplicon sequencing section. Strain-specific primers were used:

Mouse-derived 20 strains:

St.1A1: 5’-ACATGCAAGTCGAACGGGAT-3’, 5’-CTCATGTGGAACATCCGGCA-3’
St.1A11: 5’-TGCTTGCACTCACCGATAAA-3’, 5’-CGGTATTAGCACCTGTTTCCA-3’
St.1A3: 5’-GAACGGTAACAGCGAGGAAA-3’, 5’-CATCCTTTCGGATGGTTGTC-3’
St.1A6: 5’-CGAGCGAGCTTGCCTAGATG-3’, 5’-CACGTGTTACTCACCCGTCC-3’
St.1B6: 5’-CAGTGGGGACGATGGTGAC-3’, 5’-CGCTCCCTACGTATTACCGC-3’
St.C4: 5’-AAGGCCTTCGGGTCGTAAAG-3’, 5’-GCACGTAGTTAGCCGTGACT-3’
St.1H8: 5’-GGAATAGAGTGGCGAACGGG-3’, 5’-CATCCCTTGCCGTCGGG-3’
St.4A1: 5’-GAAACTGCCTGATGGAGGGG-3’, 5’-GAAGGTCCCCCACTTTGGTC-3’
St.4B7: 5’-GACGAAGCCACTTGTGGTGA-3’, 5’-ATTTCACAGACGACGCGACA-3’
St.4D1: 5’-GGCGGATTTATCTGCCGCTC-3’, 5’-CTATGCATCGTCGCCTTGGT-3’
St.4D3: 5’-GGGAAGAAGCCCCTTTTGGG-3’, 5’-TTGCGCCCTACGTATTACCG-3’
St.4H5: 5’-GATAACTCCGGGAAACCGGG-3’, 5’-ACAGCCGAAACCGTCTTTCA-3’
St.4H6: 5’-ACAGCCGAAACCGTCTTTCA-3’, 5’-TCTCCACATGGAGGGGGAAG-3’
St.8H4: 5’-ACGTATGTGGGAAAGACGGC-3’, 5’-AACCATCGTCGCCTTGGTAG-3’
St.18E8: 5’-AAAGGAGGGGAGTCAGCAAT-3’, 5’-ACAGAGTCCTCTGCTTCACCA-3’
St.19E12: 5’-CGGCACATGATACTGCGAGA-3’, 5’-TTAATGTCCAGGAACCCGCC-3’
St.21A7: 5’-GCGAAGCACTTTGATTGGAT-3’, 5’-CGCGGTCTTATGCGGTATTA-3’
St.21B5: 5’-TGAAGGCTTGCCTTTACCAG-3’, 5’-ATGTCCCGTCGATGCATTAT-3’
St.27G3: 5’-GCAAGTCGAACGAAGCATTT-3’, 5’-TGTTGTCCCCCTGTGTAAGG-3’
St.80E1: 5’-ATGCAAGTCGAGCGAACCAC-3’, 5’-TAGCGATCGTTTCCAATCGT-3’

Human-derived T19 33 strains:

St.1: 5’-TGGCGAACGGGTGAGTAATA-3’, 5’-CACCATGCAGTGTCCATACCT-3’
St.2: 5’-AGTAACGCGTGGGTAACCTG-3’, 5’-TGCGATACTGTGCGCTTATG-3’
St.3: 5’-TTCGGCCGTGTATAGAGTGG-3’, 5’-GTATTAGCCGCCGTTTCCAG-3’
St.4: 5’-AACGGGTGAGTAACGCGTAG-3’, 5’-ATCATGCGATAGCGTGGTCT-3’
St.5: 5’-GCCCTATACAGGGGGATAACA-3’, 5’-TACTGCCAGGGCTTTTCACA-3’
St.6: 5’-GAGCAACCTGCCTTTCAGAG-3’, 5’-GATTGCTCCTTTGGTTGCAG-3’
St.7: 5’-AAAGCTTGCTTTCTTTGCTG-3’, 5’-AACCATGCGGAATCATTATGC-3’
St.8: 5’-GTTTGCTTGCAACTGAAGATGG-3’, 5’-AAAGGCTATTCCGGAGTTATCG-3’
St.9: 5’-CGGGTGAGTAACACGTATCCA-3’, 5’-TGCGGAAGAATTATGCCATC-3’
St.10: 5’-CGTATCCAACCTGCCGTCTA-3’, 5’-TCATGCGGACATGTGAACTC-3’
St.11: 5’-AAGCTTGCTTTGATGGATGG-3’, 5’-TTCGAAAGGCTATCCCAGTG-3’
St.12: 5’-ACGTATCCAACCTGCCGATA-3’, 5’-CAAGACCATGCGGTCTGATT-3’
St.13: 5’-TTAGCTTGCTAAGGCCGATG-3’, 5’-CCTTTCAGAAGGCTGTCCAA-3’
St.14: 5’-GGGTGAGTAACGCGTGGATA-3’, 5’-ATCGGGAGCGTATTCGGTAT-3’
St.15: 5’-TGAGTAACGCGTGAGCAATC-3’, 5’-TCAAGAGATGCCTCCCAAAC-3’
St.16: 5’-TGGGGAATAACAGGTGGAAA-3’, 5’-GAGCGATAAATCTTTGGCAGTC-3’
St.18: 5’-CATGTGTCCGGGATAACTGC-3’, 5’-CCTTGATGGGCGCTTTAATA-3’
St.19: 5’-TGGCGAACGGGTGAGTAATA-3’, 5’-CCCTTCACCTATGCGGTCTT-3’
St.20: 5’-CTGTACCGGGGGATAACACTT-3’, 5’-CCACCGGAGTTTTTCACACT-3’
St.21: 5’-GGAAAAAGAAGAGTGGCGAAC-3’, 5’-CGGTATTAGCACCTGTTTCCA-3’
St.22: 5’-TTTTCTTTCACCGGAGCTTG-3’, 5’-CGCCTTTCAACTTTCTTCCA-3’
St.23: 5’-TGGCGAACGGGTGAGTAATA-3’, 5’-TGTCCGTACCTATGCGGTCT-3’
St.24: 5’-GATGAAGGATATGGCGACTGA-3’, 5’-GGCCTTATGCGGTATTAGCA-3’
St.25: 5’-GATTCGTCCAACGGATTGAG-3’, 5’-GCATCATGCGGTATTAGCACT-3’
St.26: 5’-AACGGGTGAGTAACACGTGAG-3’, 5’-TTGCTCCTTTTCCCTCTGTG-3’
St.27: 5’-AGTAACGCGTGGGTAACCTG-3’, 5’-ACCGGAGTTTTTCACACCAG-3’
St.28: 5’-GCGGATCTTCGGAAGTTTTC-3’, 5’-ACCGGAGTTTTTCACACCAG-3’
St.29: 5’-TGGCGAACGGGTGAGTAATA-3’, 5’-GTCCCCCTCTTTCTTCCGTA-3’
St.30: 5’-AGTAACGCGTGGGTAACCTG-3’, 5’-CCACCGGAGTTTTTCACACT-3’
St.31: 5’-AGCGATTCTCTTCGGAGAAG-3’, 5’-GCAAAAGCTTTGATACTTCT-3’
St.32: 5’-TTTCAGTGGGGGACAACATT-3’, 5’-AAATCCTTTGACCCCTGTGC-3’
St.34: 5’-TTAGTTTGCTTGCAAACTAAAG-3’, 5’-CCATGCGGTTTTAATATACC-3’
St.35: 5’-GACGGATTTCTTCGGATTGA-3’, 5’-ACCGGAGTTTTTCACACCAG-3’.

### 16S rRNA gene amplicon sequencing

Frozen SI, caecal contents and faecal pellets from mice were thawed and suspended in 500 mL TE10 (10mM Tris-HCl, 10mM EDTA) buffer containing RNase A (final concentration of 100 μg/mL, Invitrogen) and lysozyme (final concentration 3.0 mg/mL, Sigma). The suspension was incubated for 1.5 h at 37°C with gentle mixing. Then, sodium dodecyl sulfate (final concentration 1%) and proteinase K (final concentration 2 mg/mL, Nacalai) were added to the suspension and the mixture was incubated for 1 h at 55°C. High-molecular mass DNA was extracted with phenol:chloroform:isoamyl alcohol (25:24:1), precipitated with isopropanol, washed with 75% ethanol, and resuspended in 50–200 mL of TE or sterile MilliQ water. PCR was performed using 27Fmod 5’-AGRGTTTGATYMTGGCTCAG-3’ and 338R 5’-TGCTGCCTCCCGTAGGAGT-3’ to the V1-V2 region of the 16S rRNA gene. Amplicons generated from each sample (~330 bp) were subsequently purified using AMPure XP (Beckman Coulter). DNA was quantified using a Quant-iT Picogreen dsDNA assay kit (Invitrogen) and a TBS-380 Mini-Fluorometer (Turner Biosystems). The 16S metagenomic sequencing was performed using MiSeq according to the Illumina protocol. Two paired-end reads were merged using the fastq-join program based on overlapping sequences. Reads with an average quality value of <25 and inexact matches to both universal primers were filtered out. Filter-passed reads were used for further analysis after trimming off both primer sequences. For each sample, 3000 quality filter-passed reads were rearranged in descending order according to the quality value, and then the trimmed reads were uploaded to the DADA2 R package v.1.18.0 to construct amplicon sequence variants (ASVs) using the filterAndTrim function with standard parameters (maxN = 0, truncQ = 2 and maxEE = 2). Possible chimeric reads were removed with the removeBimeraDenovo function of the DADA2. Taxonomic assignment of each ASV was made via searching by similarity against the National Center for Biotechnology Information RefSeq and genome database using the GLSEARCH program.

### Bacterial whole-genome sequencing

Whole genome sequencing was performed using the Sequel II system (PacBio, Inc., CA, USA). The library was prepared using the SMRTbell Express template preparation kit v2.0 (PacBio) following DNA shearing to a target length of 10–15 kb using gTUBE (Covaris, LCC., USA). The PacBio reads were converted to HiFi reads using CCS software v.6.2.0. The HiFi reads for the mouse derived strains were assembled using both Canu v.2.1.1 and Flye v.2.9 with the following parameters: Canu (-pacbio-hifi, genomeSize=2.5M, minReadLength = 2200) and Flye (-g 2.5m, --min-overlap 2200, --pacbio-hifi). Contigs from the Canu assembly were used as genomes for analysis when consensus was reached between the two assemblers. The generated consensus contigs generated were checked for circularization to remap the HiFi reads by Minimap2 v.2.24-r1122. For human-derived strains, HiFi reads were assembled using the Hifiasm v.0.19.5-r587 with default parameters. Contigs aligned to other contigs with ≥99% identity and ≥95% coverage or higher were considered as bubble contigs. Contigs with low depth (< 5) and bubble contigs were eliminated. The genes were predicted and annotated using Bakta version 1.5.1 (ref. ^98^). Further functional annotation was performed using eggNOG-mapper (version emapper-2.1.10)^99^ based on eggNOG orthology data^100^ and a DIAMOND search algorithm^101^.

### Metabolomics analysis

For untargeted metabolomic analysis, plasma and ileal samples were suspended in 400 μL of methanol per 100 μL of plasma volume or per 100 mg of ileal contents. A 40 μL aliquot was subjected to a single-layer extraction, followed by untargeted LC–QTOF/MS analysis as previously described^102^.

For targeted metabolomic analysis focusing on bile acids, a 30 mL of plasma was mixed with 968.5 mL of 0.2 N NaOH and sonicated for 10 min in vial containing 1.5 mL of the internal standards (d4-CA, d4-GCDCA, d4-TCDCA, d4-CDCA-3S, and d4-LCA, each at 10 μM). Ileal content samples were resuspended in 20 or 2000 times the volume of water. A 100 mL of the diluted luminal suspension was homogenized in 897 mL of 0.2 N NaOH by ultrasonication for 1 hr in a screw-cap glass vial containing 3 mL of deuterium-labelled internal standards (d4-CA, d4-GCDCA, d4-TCDCA, d4-CDCA-3S, and d4-LCA, 10 mM each). After 1 hour of incubation at room temperature, the pH was adjusted to 8.0 using 12N HCl and mixed with 110 μL of 0.5 M EDTA/0.5 M Tris-HCl. The pH was checked and adjusted as needed with 17 μL of 12 N HCl. The mixture was centrifuged at 15,000 rpm for 10–20 minutes, and the supernatant was loaded onto a solid-phase extraction cartridge (Agilent Bond Elut C18, 100 mg/3 mL), preconditioned with 1 mL of methanol and 3 mL of water, repeated three times. The cartridge was washed with 1 mL of water, and the bile acids were eluted with 600 μL of 90% ethanol.

For quantification of bile acids, 2 μL of the eluted sample was injected into an LC/ESI-MS/MS system (Triple Quad 6500+ tandem mass spectrometer, equipped with an ESI probe and Exion LC AD ultra-high-pressure liquid chromatography system; SCIEX). The separation column used was InertSustain C18 (150 mm × 2.1 mm ID, 2 μm particle size; GL Sciences Inc.), maintained at 40°C. The eluent consisted of a mixture of DDW with 0.01% formic acid, 10 mM ammonium acetate, and 20% acetonitrile (mixture A), and a mixture of 30% acetonitrile and 70% methanol (mixture B). Separation was achieved using a linear gradient elution at a flow rate of 0.2 mL/min, with the following gradient profile: 30–45% B (0–14 min), 45–65% B (14–25 min), 65–75% B (25–35 min), 75–100% B (35–35 min), 100% B (35.1–40 min), 100–30% B (40–40.1 min), and 30% B (40.1–45 min). The total run time was 45 minutes. LC/ESI-MS/MS was operated with the following parameters: for positive ion MRM mode–ion spray voltage of 5,500 V, interface temperature of 400 °C, curtain gas; 25 psi, collision gas (nitrogen); 10 psi, ion source gas 1; 60 psi, and ion source gas 2; 40 psi. For the negative ion MRM mode: ion spray voltage; −4,500 V, interface temperature; 400 °C, curtain gas; 25 psi, collision gas (nitrogen); 10 psi, ion source gas 1; 60 psi, and ion source gas 2; 40 psi. Samples were obtained using Analyst software ver1.71and analysed using SCIEX OS-MQ software ver2.1.0.55343

### *In vitro* bile acid transformation

Individual bacterial strains, derived from mouse and human sources, were streaked on BHK agar plates. A single colony of each strain was inoculated into mGAM broth, with supplements added for certain strains to support growth. Overnight cultures were then diluted 100 times and incubated with 50 μM taurocholic acid (tauro-CA) at 37°C for 48 hours using mGAM or medium with different concentration of protein in some experiments. All culturing and assays were conducted in an anaerobic chamber (Coy Laboratory Products) with an atmosphere of 80% N2, 10% H2, and 10% CO2. After 48 hours, 200 μL of each bacterial suspension was collected for bile acid extraction. To each 40 μL aliquot of the suspension, 33 μL of 6.0 M NaOH, 10 μM internal standard (ISTD), and 924 μL of MilliQ water were added, followed by 10 minutes of sonication. Then, 110 μL of 0.5 M EDTA/0.5 M Tris buffer (pH 8) and 17 μL of 12 M HCl were added, adjusting pH to neutrality as needed. Samples were purified on columns pre-activated with 1 mL methanol and washed twice with 3 mL MilliQ water. After loading, samples were washed six times with 3 mL MilliQ water, and residual MilliQ was removed by adjusting the flow rate. Finally, 600 μL of 90% ethanol was applied to elute bile acids, and samples were collected in vials for bile acid quantification using LC-MS/MS.

### Metatranscriptome analysis

Extraction of total RNA from caecal contents of T19-derived 33-mix-colonized mice which fed with either CD or LPD was conducted using the NucleoSpin RNA kit (Macherey-Nagel), according to the manufacturer’s instructions. Libraries for RNA sequencing were prepared using TruSeq Stranded mRNA Library Prep (Illumina) and sequenced using HiSeq X (Illumina) or NovaSeqXPlus with the mode of 150-bp paired-end at Macrogen Japan. The sequenced paired-end reads were quality-controlled using Trimmomatic^103^ version 0.39 with “2:30:10 LEADING:20 TRAILING:20 SLIDINGWINDOW:4:20 MINLEN:30” options. The quality-controlled reads were mapped to concatenated reference genome sequence of T19-derived 33 stains using STAR version 2.7.10b with “outFilterMultimapNmax: 20 alignIntronMax: 1” options. Aligned sorted bam files were generated using samtools version 1.19.2 and visualized using Integrative Genomics Viewer version 2.17.4. The read counts for each gene were obtained using featureCounts^104^ Rsubread 2.12.3 with “countMultiMappingReads=TRUE,fraction=TRUE” options and further normalized by bacterial abundance using absolute DNA levels of each strain and total bacteria which were quantified by qPCR using 16S rRNA primers as previously described^105^. The differential expression analysis was performed using DESeq2 (ref. ^106^) version 1.38.3 with setting cutoff adjusted p-value<0.05. Upregulated genes of St.3, St.4, St.14 and St.31 were used for enrichment analysis using enricher function of clusterProfiler version1.38.3 (ref. ^107^) against all KEGG pathways which are included in 1. Metabolism, 2. Genetic Information Processing, 3. Environmental Information Processing and 4. Cellular Processes.

### Generation of *Bilophila* sp. 4_1_30 (St.14) mutants

The *nrfA* (gene id: OMIHGE_02465) gene deletion mutant of *Bilophila* sp. 4_1_30 (St.14) was generated in a similar manner for *Nitratidesulfovibrio vulgaris* “marker-exchange” mutant as described in previous reports^108,109^, as shown in **Supplementary Fig.1**. First, a uracil phosphoribosyltransferase (*upp,* gene id: OMIHGE_01720) deletion mutant (Δ*upp*) was generated and then, marker-exchange mutant (Δ*upp*, Δ*nrfA::*(*cat upp*)) was generated one the parent Δ*upp* strain by electroporation-mediated plasmid introduction and selection with 5-fluorouracil (5-FU) and chloramphenicol. To generate Δ*upp* mutant, approximately 0.9-kb sequences flanking the coding region were amplified by PCR (PCR primers used in this study are listed in **Supplementary Table 6**) and assembled into the PstI and SalI sites of the pBluescriptIISK+ using the HiFi DNA Assembly (NEB) as per the manufacturer’s protocol to make *upp* depletion cassette. The electroporation was carried out in a total volume of 80 mL with an ELEPO21 Electroporator (Nepa Gene) in 1-mm gapped electroporation cuvettes at 1500–2000 V with default setting. The cells were allowed to recover in 1 mL of mGAM broth supplemented with 1 % taurine overnight at 37°C and 50 mL of the bacterial suspension was plated on mGAM agar containing 1% taurine and 40 mg/ml 5-FU for 4 days in the anaerobic chamber. 5-FU resistant colonies were selected and the deletion of the *upp* gene was verified by PCRs and by culturing on mGAM agar containing 40 mg/mL 5-FU. To generate marker-exchange mutant of *nrfA,* targeting plasmid was constructed to contain the pUC origin of replication, the ampicilin resistance gene (for selection of *E. coli* DH5a), the 1,5-kbp region upstream of the *nrfA* gene, a chloramphenicol resistance gene (Chloramphenicol acetyltransferase; cat) and the promoter, upp under the kanamycin resistance gene promoter (PaphIIa), and a 1,5-kbp region downstream of the *nrfA* gene. A successful transformation of the marker-exchange plasmid into a *Bilophila* sp. 4_1_30 (St.14) lacking the upp gene will replace the *nrfA* gene with the Chloramphenicol resistance and upp genes via a double-homologous recombination event. This would result in a transformant resistant to chloramphenicol and sensitive to 5-FU. Transformation was accomplished by electroporation with same condition used for generation of Δ*upp* mutant. Cells were allowed to recover overnight in mGAM broth supplemented with 1 % taurine and 50 mL of the bacterial suspension was plated on mGAM agar containing 1% taurine and 80 mg/ml chloramphenicol for 5 days in the anaerobic chamber. Chloramphenicol resistant and 5-FU sensitive colonies were selected and the deletion of the *nrfA* gene was verified by PCRs and Sanger sequencing. In Figure 7 i, j and Extended Data Figure 17, the Δ*upp*, Δ*nrfA::*(*cat upp*) strain was referred to as the Δ*nrfA,* and the parental Δ*upp* strains was referred to as the wild-type *nrfA* strain.

### Protein homology search for 7α-hydroxysteroid dehydrogenase

We used mmseq2 (version ffb05619cadadd8655b8719818ed566caaa6d0a6) to align 3 experimentally validated 7α-hydroxysteroid dehydrogenase protein sequences to all proteins from *Romboutsia timonensis, Adlercreutzia equolifaciens,Bilophila* sp. 4_1_30, and *Anaerofustis stercorihominis*. The protein from each isolate that aligned best to each reference protein was plotted in a heatmap using the pheatmap package.

### Protein homology search to NrfA

We annotated all proteins in each of the 33 human isolates using eggnog-mapper (version 2.1.12). We ran each protein annotated to NrfA through signalP to discovery the presence or absence of a signal sequence, and the specific type of signal sequence. UHGG version 2.0.2 reference genome eggnog annotations were downloaded from https://ftp.ebi.ac.uk/pub/databases/metagenomics/mgnify_genomes/human-gut/v2.0.2/species_catalogue/. GTDB-Tk version 2.4.0 was used to identify and align 120 bacterial genes across all UHGG reference genomes. FastTree with the parameter “-lg” was used to create the phylogenetic tree. All proteins in UHGG annotated to NrfA by eggnog annotation were also run through signalP to annotate the signal sequence.

To find nitrite reductase proteins in *Taurnivorans muris,* we annotated all proteins using Gapseq (version 1.3.1 690bece9) using default parameters as well CLEAN (Enzyme Function Prediction using Contrastive Learning).

### Statistical analysis

Statistical analyses were performed using GraphPad Prism software version 9, Strand NGS v.2.7 and DAVID (for bulk RNAseq analysis) or R (version 4, for metatranscriptome analysis). Specifically, the two-tailed unpaired Student’s t-test (parametric) was used for all comparisons between two groups. One-way analysis of variance (ANOVA) followed by Benjamini–Hochberg correction for multiple comparisons was used for all comparisons between three or more groups, except for bile acids concentration comparisons, which were analyzed using the two-tailed Mann-Whitney test for each comparison of two groups. For time-course analysis of body weight, relative gene expression, and blood glucose, two-way ANOVA with Bonferroni’s test (comparisons between two groups, parametric) or Benjamini–Hochberg correction (comparisons between three or more groups, parametric) was used.

## Data Availability

Genome sequences of the mouse-derived 20 and human (T19)-derived 33 strains are deposited in the DNA Data Bank of Japan under BioProject PRJDB19530. Data of bulk RNAseq, snRNAseq and metatranscriptome will be deposited in the DNA Data Bank of Japan as well.
